# Inhibition of p38 MAPK sensitizes tumour cells to cisplatin-induced apoptosis mediated by reactive oxygen species and JNK

**DOI:** 10.1002/emmm.201302732

**Published:** 2013-09-24

**Authors:** Lorena Pereira, Ana Igea, Begoña Canovas, Ignacio Dolado, Angel R Nebreda

**Affiliations:** 1 Institute for Research in Biomedicine (IRB Barcelona)Barcelona, Spain; 2 Institució Catalana de Recerca i Estudis Avançats (ICREA)Barcelona, Spain

**Keywords:** apoptosis, cisplatin, JNK, p38 MAPK, ROS

## Abstract

The p38 MAPK pathway is an important regulator of many cellular responses. It is well established that p38 MAPK signalling negatively regulates epithelial cell transformation, but enhanced p38 MAPK activity has been also correlated with bad clinical prognosis in some tumour types. Here, we provide genetic and pharmacological evidence showing that p38 MAPK inhibition cooperates with the chemotherapeutic agent cisplatin to kill tumour cells. We show that p38 MAPK inhibition results in ROS upregulation, which in turn activates the JNK pathway via inactivation of phosphatases, sensitizing human tumour cells to cisplatin-induced apoptosis. Using a mouse model for breast cancer, we confirm that inhibition of p38 MAPK cooperates with cisplatin treatment to reduce tumour size and malignancy *in vivo*. Taken together, our results illustrate a new function of p38 MAPK that helps tumour cells to survive chemotherapeutic drug treatments, and reveal that the combination of p38 MAPK inhibitors with cisplatin can be potentially exploited for cancer therapy.

## INTRODUCTION

Regulation of the redox state is essential to maintain cellular homeostasis. A number of reports have shown that elevated reactive oxygen species (ROS) can promote malignant transformation in fibroblasts (Du et al, 2009; Suh et al, 1999; Zimmerman & Cerutti, 1984), and dysregulated ROS production is also associated with enhanced tumourigenicity in tumour epithelial cells (Dolado et al, 2007). However, the pro-tumourigenic effect of the accumulation of intracellular ROS can turn out to be a double-edged sword, since high ROS levels may lower the apoptotic threshold for cytotoxicity (Trachootham et al, 2009; Wang & Yi, 2008). Therefore, redox regulation plays an important role in tumour cell survival. Consistent with this idea, some cytostatic treatments can kill transformed cells more efficiently when combined with pharmacologic agents that deplete reducing metabolites or enhance ROS production (Engel & Evens, 2006). For example, the ROS-producing agent emodin increases the sensitivity of cancer cells to platinum drugs (Wang et al, 2010). Likewise, administration of cisplatin together with the reducing metabolite-depleting agent buthionine sulphoximine (BSO), overcomes the cisplatin resistance of MCF7 breast cancer cells (Rudin et al, 2003). A recent publication has also shown how the different ROS content of transformed and normal cells can be used to design therapies that would preferentially target transformed cells (Raj et al, 2011).

Cisplatin is a DNA damage-inducing agent that is widely used for cancer treatment. Its anti-tumoural properties are mainly based on the induction of DNA cross-links (Brozovic & Osmak, 2007). An important limitation for the successful use of cisplatin in chemotherapy is the development of resistance (Rottenberg et al, 2007). Experiments with cancer cell lines have associated sensitivity to cisplatin with activation of p38 mitogen-activated protein kinase (MAPK) signalling. However, cancer cell lines with high basal levels of p38 MAPK activity tend to be more resistant to cisplatin, suggesting that the inability to further activate p38 MAPK in response to cisplatin correlates with resistance (Galan-Moya et al, 2011). The mechanism by which p38 MAPK signalling could impinge on cisplatin sensitivity or resistance has not been elucidated.

There are four p38 MAPKs, with p38α and p38β having similar substrate specificities. However, whereas p38α is the most abundant family member in most cell types, p38β is usually expressed in a more restricted manner and at lower levels (Cuadrado & Nebreda, 2010). Experiments using genetically modified mice have shown that p38α and p38β play overlapping functions during embryo development (del Barco Barrantes et al, 2011). The p38α signalling pathway is an important regulator of stress responses, and there is also evidence that p38α can function as a tumour suppressor by inducing cell cycle arrest, differentiation and apoptosis (Wagner & Nebreda, 2009). However, this function seems to be mainly operative at the onset of cellular transformation (Dolado et al, 2007; Hui et al, 2007; Ventura et al, 2007), as enhanced p38 MAPK phosphorylation has been correlated with poor overall survival in patients with HER-2 negative breast cancer (Esteva et al, 2004) or with hepatocellular carcinoma (Wang et al, 2012). Furthermore, p38 MAPK overexpression or overactivation has been reported in transformed follicular lymphomas (Elenitoba-Johnson et al, 2003) and in thyroid neoplasms (Pomerance et al, 2006). The potential pro-tumourigenic roles of p38α signalling are not only based on correlations with bad prognosis in cancer, as there is evidence that this pathway may contribute to the survival or proliferation of cancer cell lines from different origins, including breast (Chen et al, 2009), colorectal (Chiacchiera & Simone, 2009), prostate (Ricote et al, 2006) or skin (Schindler et al, 2009).

An important function of the p38α pathway is to facilitate cell survival in response to stress (Thornton & Rincon, 2009), for instance by inducing cell cycle arrest when cells are exposed to osmotic shock or oxidative stress (Joaquin et al, 2012). Here, we show that p38α and to a lesser extent p38β play a key role in the viability of cancer cells treated with the chemotherapeutic agent cisplatin. We demonstrate that p38 MAPK inhibition results in ROS-dependent upregulation of the JNK pathway, which in turn potentiates cisplatin-induced apoptosis. Importantly, we provide *in vivo* evidence supporting that p38 MAPK inhibition cooperates with cisplatin treatment to reduce the size and malignancy of breast tumours in mice.

## RESULTS

### Inhibition of p38 MAPK signalling sensitizes to apoptosis by activating the JNK pathway

**Figure 1:**
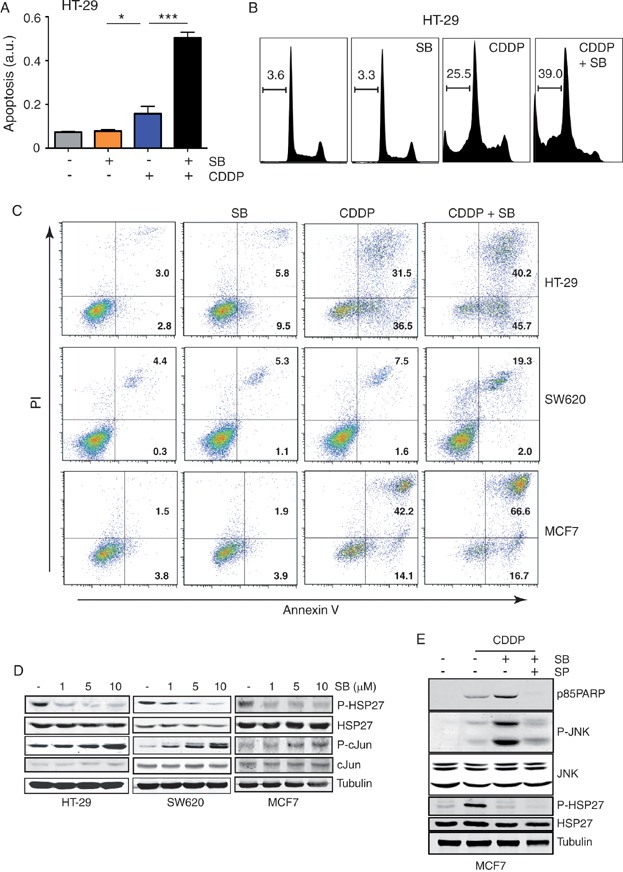
Inhibition of p38 MAPK in cancer cells activates JNK and sensitizes to apoptosis Source data is available for this figure in the Supporting Information. HT-29 cells were incubated with SB203580 (SB, 10 μM) overnight followed by cisplatin (CDDP, 100 μM) for 8 h, as indicated. Apoptosis was measured with the Cell Death Detection ELISA kit. ***p < 0.0001, *p < 0.05.HT-29 cells were treated as in (A) and the apoptotic sub G0/G1 population (indicated by a solid line) was analysed by flow cytometry.HT-29, SW620 and MCF7 cells were incubated with SB203580 (SB, 10 μM) for 2 h followed by treatment with cisplatin (CDDP, 100 μM) for 24 h, and then were stained with propidium iodide (PI) and Annexin V. The percentages of apoptotic cells are indicated.HT-29, SW620 and MCF7 cells were treated with increasing concentrations of SB203580 (SB, 1–10 μM) for 6 h and total cell lysates were analysed by immunoblotting with the indicated antibodies.MCF7 cells were treated overnight with SB203580 (SB, 10 μM) alone or in combination with SP600125 (SP, 20 μM) for 1 h, followed by 8 h with cisplatin (CDDP, 100 μM). Total cell lysates were analysed by immunoblotting with the indicated antibodies.

To determine the role of p38 MAPK signalling in the survival of cancer cells exposed to chemotherapeutic agents, we treated human breast and colon cancer cell lines with cisplatin together with SB203580, a chemical inhibitor of p38α and the related family member p38β. The combination of cisplatin with SB203580 significantly potentiated the induction of apoptosis in HT-29 colon cancer cells compared to cisplatin alone, as determined by the release of DNA oligonucleosomes (Fig 1A) or by the percentage of cells that were in the subG0/G1 phase of the cell cycle (Fig 1B). Annexin V staining confirmed the enhanced cisplatin-induced apoptosis in response to p38 MAPK inhibition in colon and breast cancer cells (Fig 1C).

Several reports indicate that p38 MAPK signalling can negatively regulate the JNK pathway in different contexts, mainly in non-transformed cells (Perdiguero et al, 2007; Wagner & Nebreda, 2009). Since JNK signalling plays an important role in apoptosis induction (Davis, 2000), we investigated whether this pathway was implicated in the enhanced apoptosis observed upon p38 MAPK inhibition. We found that inhibition of p38 MAPK signalling with SB203580, as shown by the reduced phosphorylation of Hsp27, resulted in enhanced activation of the JNK pathway in three different human cancer cell lines from breast and colon origin (Fig 1D and Supporting Information Fig S1A). In agreement with the known role of the JNK pathway in cisplatin effects (Brozovic & Osmak, 2007), we found that the JNK chemical inhibitor SP600125 impaired the enhanced apoptosis observed in cisplatin-treated cancer cells when p38 MAPK was inhibited, as determined by the reduced levels of caspase-cleaved poly(ADP-ribose) polymerase (p85PARP) (Fig 1E). These results indicate a functional interplay between both signalling cascades in cancer cells, with the JNK pathway mediating the enhanced apoptosis induced by cisplatin upon p38 MAPK inhibition.

To rule out possible off-target effects, we used another p38 MAPK inhibitor. We chose PH-797804, a potent inhibitor of p38α and p38β that is currently in clinical trials (Goldstein et al, 2010; Hope et al, 2009). We confirmed that cancer cells treated with PH-797804 showed increased cell death in response to cisplatin, as determined by Annexin V staining (Supporting Information Fig S1B). Western blot analysis also confirmed activation of the JNK pathway and increased levels of processed p85PARP in cancer cells treated with cisplatin and PH-797804 (Supporting Information Fig S1C).

**Figure 2:**
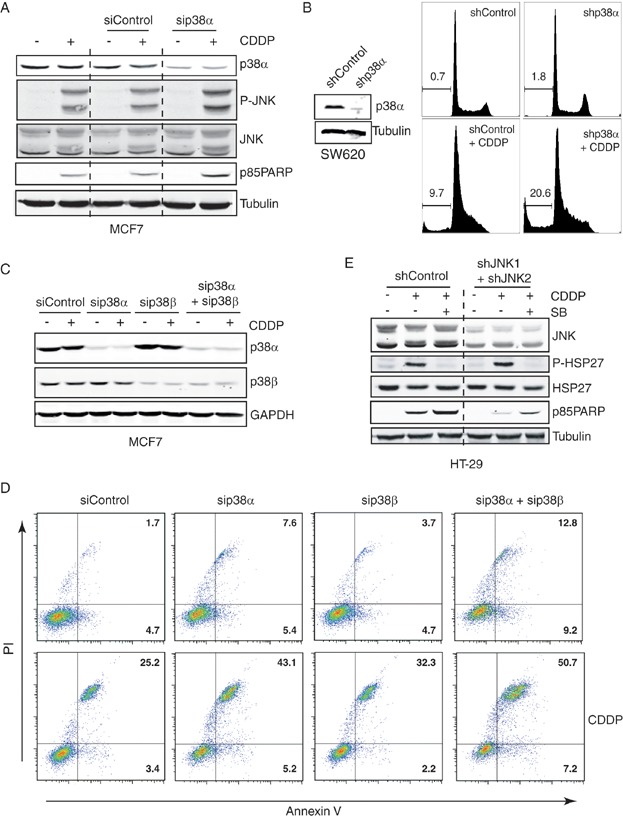
p38 MAPK downregulation activates JNK and increases apoptosis in cancer cells treated with cisplatin Source data is available for this figure in the Supporting Information. MCF7 cells were incubated for 48 h with a siRNA against p38α (#2) or a scrambled siRNA as a control and then treated for 8 h with cisplatin (CDDP, 100 μM). Total cell lysates were analysed by immunoblotting with the indicated antibodies.SW620 cells were infected with lentiviruses expressing shRNAs against p38α (shp38α) or a non-targeting control (shControl). After 1 week of puromycin selection, pools of cells were treated for 24 h with cisplatin (CDDP, 100 μM) and the apoptotic sub G0/G1 population was quantified by flow cytometry. In parallel, samples were analysed by immunoblotting to confirm the downregulation of p38α.MCF7 cells were incubated for 48 h with siRNAs against p38α (#2), p38β, or both together, or with a scrambled siRNA as a control, and then treated for 8 h with cisplatin (CDDP, 100 μM). Total cell lysates were analysed by immunoblotting with the indicated antibodies.MCF7 cells were treated as in (C) and incubated with SB203580 (SB, 10 μM) for 2 h followed by treatment with cisplatin (CDDP, 100 μM) for 24 h, and then stained with propidium iodide (PI) and Annexin V. The percentages of apoptotic cells are indicated.HT-29 cells were infected with shRNAs against JNK1 and JNK2 or a non-targeting control. After 1 week of puromycin selection, pools of cells were incubated overnight in the presence or absence of SB203580 (SB, 10 μM) and then treated for 8 h with cisplatin (CDDP, 100 μM). Total cell lysates were analysed by immunoblotting with the indicated antibodies.

The above results were validated using RNAi. Consistent with the high expression levels of p38α in most cell types, we confirmed that RNAi-mediated downregulation of p38α resulted in enhanced levels of JNK phosphorylation as well as increased cisplatin-induced apoptosis in both breast and colon cancer cells (Fig 2A and B). Since the chemical compounds SB203580 and PH-797804 can inhibit both p38α and p38β, we also analysed the potential contribution of p38β. We found that siRNA-mediated knockdown of p38β enhanced cisplatin-induced cell death and JNK phosphorylation, although the effect was smaller than in the case of p38α knockdown (Fig 2C and D and Supporting Information Fig S2A). Moreover, knockdown of both p38 MAPK family members resulted in higher levels of cell death than the individual knockdowns (Fig 2D). The results indicate that p38α and p38β are both implicated in the cisplatin response, with p38α playing a major role. Next, we downregulated JNK1 and JNK2 with shRNAs, which confirmed a key role for this signalling pathway in the apoptosis induced by cisplatin in cancer cells. Interestingly, JNK1 and JNK2 downregulation decreased the enhanced apoptosis induced by cisplatin when p38 MAPK signalling was inhibited (Fig 2E). These results were confirmed using a siRNA that simultaneously downregulates JNK1 and JNK2 and measuring apoptosis by Annexin V staining (Supporting Information Fig S2B). In contrast, downregulation of only JNK1 or JNK2 did not significantly affect cisplatin-induced apoptosis in cancer cells (Supporting Information Fig S2C). These results indicate that JNK1 and JNK2 play redundant roles and that both are involved in the sensitization to cisplatin-induced cell death upon p38 MAPK inhibition.

### Increased ROS levels mediate JNK activation upon p38 MAPK inhibition

**Figure 3:**
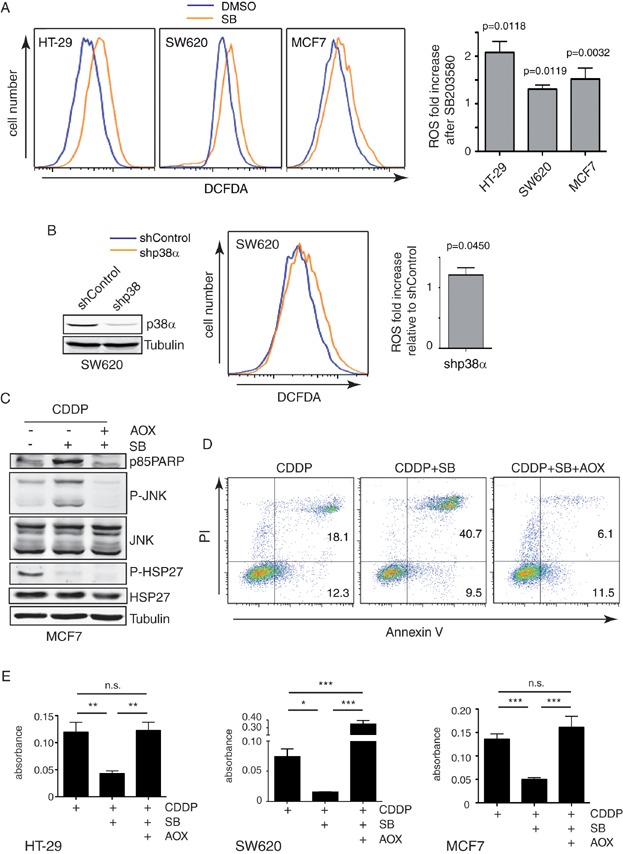
Inhibition of p38 MAPK activates JNK through ROS upregulation Source data is available for this figure in the Supporting Information. The indicated cancer cells were incubated overnight either with DMSO or SB203580 (SB, 10 μM) and ROS levels were measured with DCFDA by flow cytometry. Fold increase of ROS levels compared to the same cells treated with DMSO is indicated in the bar diagram.SW620 cells were infected with lentiviruses expressing shRNAs against p38α or a non-targeting control. Pools of cells were selected with puromycin for 1 week and basal ROS levels were measured with DCFDA by flow cytometry. Samples were also analysed by immunoblotting to confirm the downregulation of p38α.MCF7 cells were pre-treated for 1 h with a mixture of NAC and GSH antioxidants (AOX), followed by overnight incubation with SB203580 (SB, 10 μM) and then treated for 8 h with cisplatin (CDDP, 100 μM). Total cell lysates were analysed by immunoblotting with the indicated antibodies.MCF7 cells were treated as in (C) and cell death was quantified by staining with propidium iodide (PI) and Annexin V.For clonogenic assays, cells were pre-treated for 1 h with a mixture of NAC and GSH antioxidants (AOX), followed by overnight incubation with SB203580 (SB, 10 μM), and then treated for 1 h with cisplatin (CDDP). Colonies were dissolved in methanol and the absorbance reads (540 nm) are represented in the bar diagrams. ***p < 0.0001, **p < 0.001, *p < 0.05, ns = *p* > 0.05.

It has been reported that JNK signalling can be activated by ROS (Shen & Liu, 2006) and high endogenous ROS levels have been correlated with activation of the JNK pathway in human cancer cells (Dolado et al, 2007). Moreover, there is evidence that p38 MAPK signalling sometimes impairs ROS accumulation (Dolado et al, 2007; Gutierrez-Uzquiza et al, 2012) and p38α downregulation in ovarian tumours has been associated with an oxidative stress gene expression signature (Mateescu et al, 2011). Therefore, we investigated whether inhibition of p38 MAPK signalling might result in increased ROS levels, which in turn would activate the JNK pathway. We found that SB203580 produced a modest but consistent increase in the endogenous ROS levels of different human cancer cell lines (Fig 3A), which was time-dependent (Supporting Information Fig S3A). Similar results were observed upon shRNA-mediated downregulation of p38α (Fig 3B).

To investigate the significance of the observed changes in ROS, we measured the effect of p38 MAPK inhibition on the overall oxidation state of cellular proteins using a chemical derivatization protocol. These experiments showed that the enhanced ROS levels induced by incubation with SB203580 resulted in higher levels of protein oxidation in the cancer cells (Supporting Information Fig S3B). Moreover, incubation with antioxidants rescued the sensitization to cisplatin-induced apoptosis produced by SB203580, as detected either by western blotting (Fig 3C) or by Annexin V staining (Fig 3D). Similar results were obtained using PH-797804 (Supporting Information Fig S3C). Importantly, clonogenic assays confirmed that p38 MAPK inhibition reduced the survival of cisplatin-treated cancer cells, whereas antioxidant treatment compensated for the effect of p38 MAPK inhibition resulting in higher overall survival of the cancer cell population (Fig 3E and Supporting Information Fig S4A). These results were confirmed using two different siRNAs to downregulate p38α (Supporting Information Fig S4B). Taken together, the results indicate that ROS induced upon p38 MAPK inhibition are responsible for the upregulation of the JNK pathway, which in turn induces tumour cell death.

**Figure 4:**
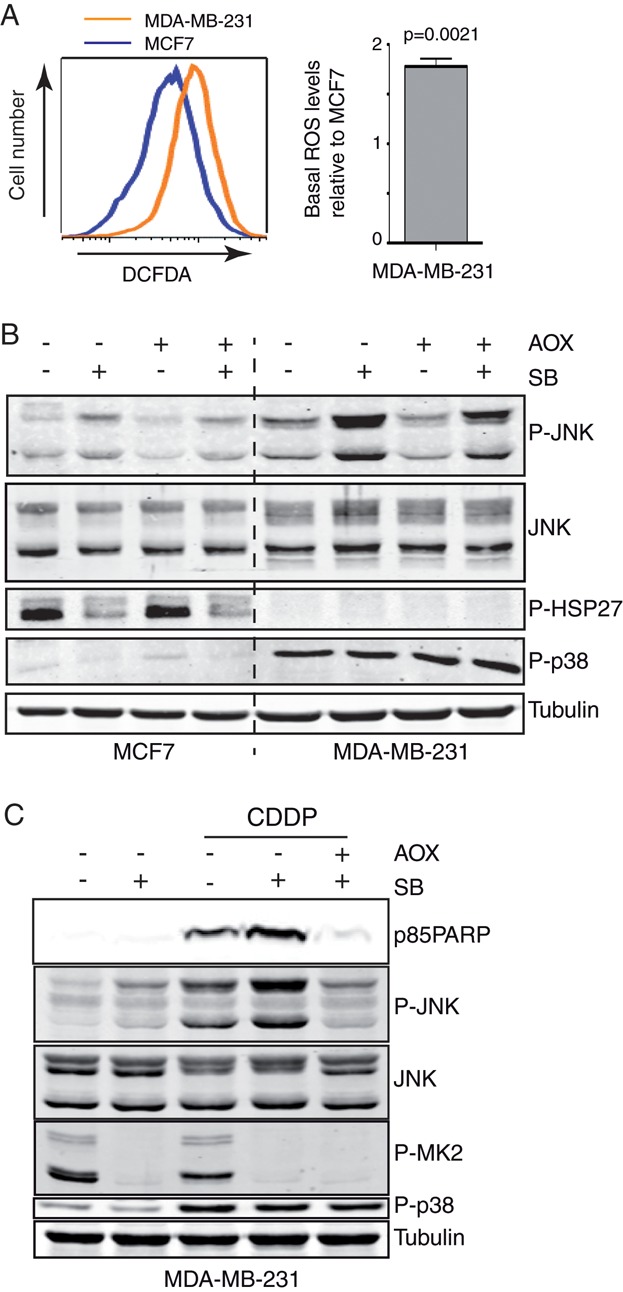
p38 MAPK inhibition sensitizes cancer cells to apoptosis regardless of their basal ROS levels Source data is available for this figure in the Supporting Information. Endogenous ROS levels of MCF7 and MDA-MB-231 human breast cancer cells were measured with DCFDA by flow cytometry. Fold increase of ROS levels compared to MCF7 cells is represented in the bar diagram.Cells were pre-treated for 1 h with a mixture of NAC and GSH antioxidants (AOX), followed by overnight incubation in the presence or absence of SB203580 (SB, 10 μM). Total cell lysates were analysed by immunoblotting with the indicated antibodies.MDA-MB-231 cells were pre-treated for 1 h with a mixture of NAC and GSH antioxidants (AOX), followed by overnight incubation with SB203580 (SB, 10 μM) and then treated with cisplatin (CDDP, 100 μM) for 8 h. Total cell lysates were analysed by immunoblotting with the indicated antibodies.

Since ROS upregulation potentiated cisplatin-induced apoptosis, we wondered whether cancer cells that have high basal levels of ROS would be also sensitized to apoptosis upon p38 MAPK inhibition. To address this question, we selected the breast cancer cell line MDA-MB-231, which has higher levels of basal ROS than MCF7 cells (Fig 4A). We found that MDA-MB-231 cells also showed JNK hyperactivation in response to p38 MAPK inhibition, and this effect was impaired by antioxidants (Fig 4B). Moreover, inhibition of p38 MAPK induced a time-dependent accumulation of ROS in MDA-MB-231 cells (Supporting Information Fig S5). In agreement with these observations, p38 MAPK inhibition further activated JNK signalling and enhanced apoptosis in cisplatin-treated MDA-MB-231 cells in a ROS-dependent manner (Fig 4C). These results indicate that the mechanism linking the inhibition of p38 MAPK with ROS accumulation, JNK activation and apoptosis induction is probably relevant to different types of cancer cells, regardless of their basal ROS content.

### Inhibition of p38 MAPK downregulates antioxidant enzymes

**Figure 5:**
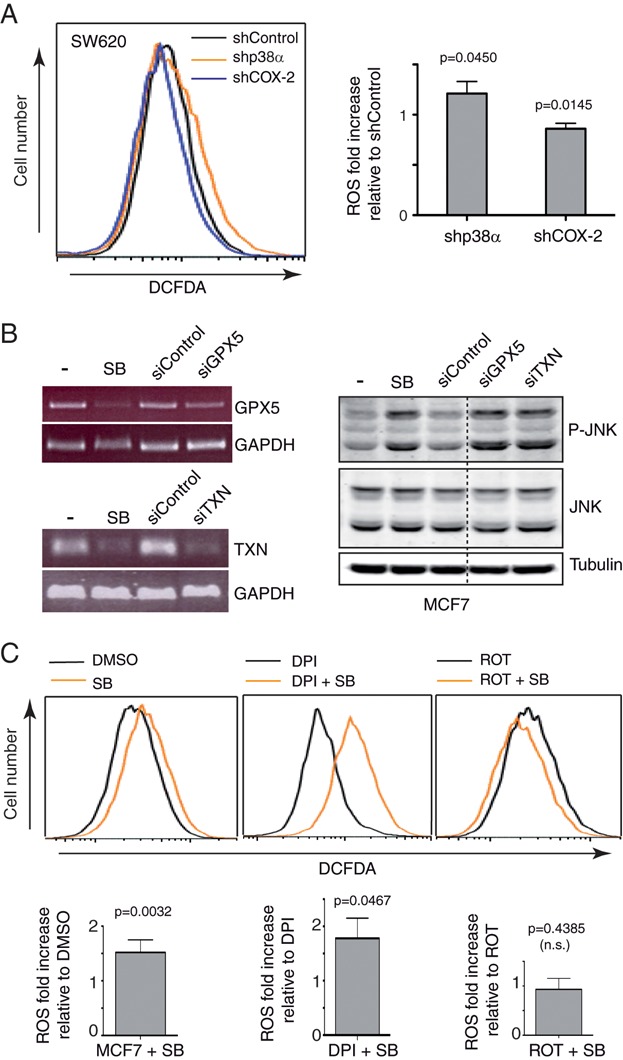
Downregulation of antioxidant enzymes induces JNK activation Source data is available for this figure in the Supporting Information. SW620 cells were infected with lentiviruses expressing shRNAs against *PTGS2* (shCOX-2) and p38α (shp38α) or a non-targeting control (shControl). Pools of cells were selected with puromycin for 1 week and levels of ROS were measured with DCFDA by flow cytometry. Fold increase of ROS levels compared to shControl cells is represented in the bar diagram.MCF7 breast cancer cells were transfected with siRNAs against *GPX5*, *TXNDC2* or a scramble control. The expression levels of the indicated mRNAs were determined by semi-quantitative RT-PCR (left panel). Total cell lysates were analysed by immunoblotting with the indicated antibodies. All samples were run on the same gel but an irrelevant lane was removed from the blot (right panel).MCF7 cells were pretreated for 1 h with DPI (2.5 μM) or rotenone (ROT, 0.25 μg/ml), incubated for 6 h with DMSO or SB203580 (SB, 10 μM), and ROS levels were measured with DCFDA by flow cytometry. Bar diagrams show ROS fold increases compared to their respective controls.

To characterize the mechanism by which inhibition of p38 MAPK can increase the levels of ROS, we used an array of 84 genes, which have been associated with oxidative stress regulation (see Materials and Methods Section). We identified eight genes with potential antioxidant functions whose expression was downregulated more than 1.5-fold in cancer cells incubated with SB203580. Re-examination of these candidate genes with custom primers showed that three genes were significantly downregulated upon p38 MAPK inhibition both in breast and colon cancer cells (Supporting Information Table S1). One of these genes was *PTGS2*, which encodes the anti-inflammatory protein Cyclooxygenase 2 (COX-2). This was described as an antioxidant gene in the array but we were not aware of clear evidence linking COX-2 to antioxidant activity. Using shRNAs, we found no evidence for a major anti-oxidant role of COX-2 in cancer cells; if anything there was a slight decrease in ROS levels upon COX-2 downregulation. As a control, shRNA-mediated downregulation of p38α in the same cancer cells showed the expected increase in ROS production (Fig 5A). Therefore, the regulation of COX-2 expression by p38 MAPK does not seem to impinge on the ROS levels of cancer cells.

The other two genes identified were *GPX5* and *TXNDC2*, which encode a glutathione peroxidase and a thioredoxin, respectively, two well-known enzymes with antioxidant activity. Interestingly, siRNA-mediated downregulation of *GPX5* or *TXNDC2* sufficed to enhance the basal JNK activity in MCF7 cells (Fig 5B). This indicates that *GPX5* and *TXNDC2* are both good candidates to mediate the upregulation of ROS observed upon p38 MAPK inhibition in cancer cells. Thus, reduced expression of these antioxidant enzymes after inhibition of p38 MAPK will result in ROS upregulation and increased levels of phospho-JNK.

We also investigated the possible contribution of NADPH oxidases (Nox) or the mitochondria to ROS generation upon p38 MAPK inhibition. We observed that the Nox inhibitor diphenyleneiodonium chloride (DPI) did not affect the production of ROS after p38 MAPK inhibition but rotenone, an inhibitor of the mitochondrial pathway, was able to block it (Fig 5C). This indicates that deregulation of mitochondrial activity might also contribute to the production of ROS when p38 MAPK is inhibited in cancer cells.

A recent report has proposed that p38α regulates ROS production in MEFs through the antioxidant enzymes catalase and superoxide dismutase (Gutierrez-Uzquiza et al, 2012). However, inhibition of p38 MAPK did not affect the expression of these enzymes in different cancer cell lines (Supporting Information Fig S6), suggesting that regulation of ROS production by p38 MAPK may involve different mechanisms in non-transformed and tumour cells.

### Inactivation of JNK phosphatases by increased ROS levels

**Figure 6:**
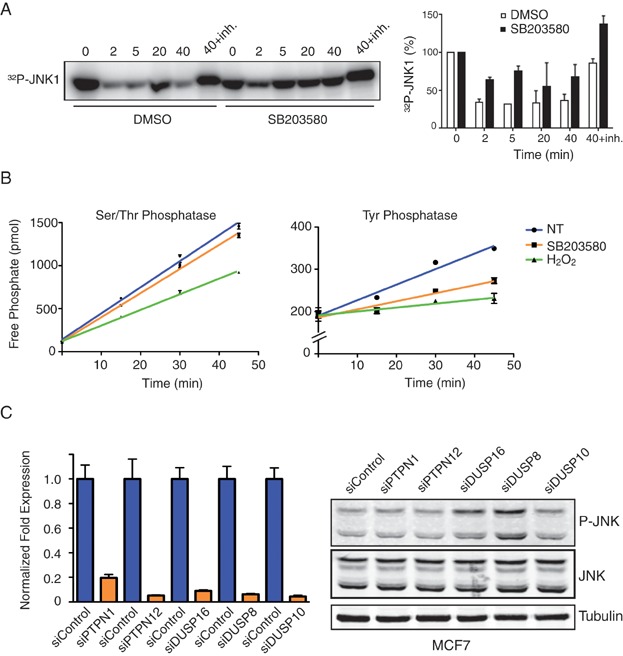
Increased ROS levels inactivate JNK phosphatases Source data is available for this figure in the Supporting Information. Recombinant GST-JNK1 protein was incubated with MKK7 and ^32^P-γ-ATP, affinity purified and then incubated for the indicated times (0–40 min) with total cell lysates obtained from SW620 cells treated or not with SB203580 (SB, 10 μM). As a control, phosphatase inhibitors were added to the SW620 cell lysates (40 + inh). The amount of phosphorylated JNK1 was visualized by autoradiography and was quantified relative to the amount present at time 0 (bar diagram).SW620 cells were incubated with SB203580 (SB, 10 μM) overnight or with H_2_O_2_ (5 mM) for 1 h and the Ser/Thr or Tyr phosphatase activities were measured in total cell lysates.MCF7 cells were transfected with siRNAs against PTPN1, PTPN12, DUSP16, DUSP8, or DUSP10 and 48 h later total cell lysates were analysed by immunoblotting with the indicated antibodies (right panel). Downregulation of the target genes was confirmed by qRT-PCR (left panel).

Previous reports have shown that ROS can lead to the inhibition of phosphatases, since their catalytic cysteine is susceptible to inactivation by oxidation (Kamata et al, 2005; Teng et al, 2007). Thus, we investigated whether the activation of JNK observed upon p38 MAPK inhibition was due to the ROS-mediated inactivation of phosphatases. We incubated *in vitro*-phosphorylated recombinant JNK1 protein with lysates of cancer cells, which had been treated or not with SB203580. We observed that the lysates of SB203580-treated cells were less efficient at dephosphorylating JNK1, suggesting that JNK phosphatases were less active in cells where p38 MAPK was inhibited, probably due to their higher levels of ROS. As a control, we confirmed that treatment with phosphatase inhibitors impaired JNK1 dephosphorylation by the cell lysates (Fig 6A).

To address what type of phosphatases was affected by the ROS produced upon p38 MAPK inhibition, we measured the Tyr or Ser/Thr phosphatase activities of cells treated or not with SB203580. We observed that the rate of dephosphorylation of Tyr phosphopeptides was significantly reduced upon p38 MAPK inhibition, whereas dephosphorylation of Ser/Thr phosphopeptides was much less affected. As a control, treatment with H_2_O_2_ significantly reduced both types of phosphatase activities (Fig 6B). Next, we focused on phosphatases that are susceptible to ROS inactivation and that have been reported to negatively regulate JNK activity, such as PTP1B, PTP-PEST, DUSP8, DUSP10 and DUSP16 (Keyse, 2008; Wu et al, 2008). Interestingly, these phosphatases have been reported to be dysregulated in breast and colon cancer cells (Labbe et al, 2012). Using siRNAs, we found that knockdown of either DUSP8 or DUSP16 enhanced the basal phosphorylation of JNK in MCF7 cells, although the effect was stronger upon DUSP8 downregulation (Fig 6C). This result indicates that DUSP8 and DUSP16 are phosphatases that control the basal activity of JNK, and whose inactivation due to increased ROS levels could explain the observed activation of JNK induced after p38 MAPK inhibition in MCF7 cells. However, DUSP8 and DUSP16 did not seem to play a major role in the regulation of basal JNK phosphorylation levels in other cancer cell lines (Supporting Information Fig S7), suggesting that different ROS-regulatable phosphatases might control the basal JNK activity in different types of tumour cells.

We considered the possibility that inhibition of p38 MAPK could impinge on upstream activators of the JNK pathway, but were not able to detect changes in the phosphorylation levels of MKK4 and MKK7 in these cancer cell lines. We also used a chemical inhibitor of ASK1, a MAP3K that mediates oxidative stress-induced phosphorylation of JNK activators, and found that this inhibitor impaired the activation of JNK induced by UV (Supporting Information Fig S8A). However, the ASK1 inhibitor affected neither JNK activation nor the enhanced apoptosis induced by cisplatin upon p38 MAPK inhibition (Supporting Information Fig S8B). Taken together, our results suggest that phosphatase inhibition plays a key role in the ROS-induced upregulation of JNK activity triggered by the inhibition of p38 MAPK.

### p38 MAPK inhibition cooperates with cisplatin to reduce breast tumour size in mice

**Figure 7:**
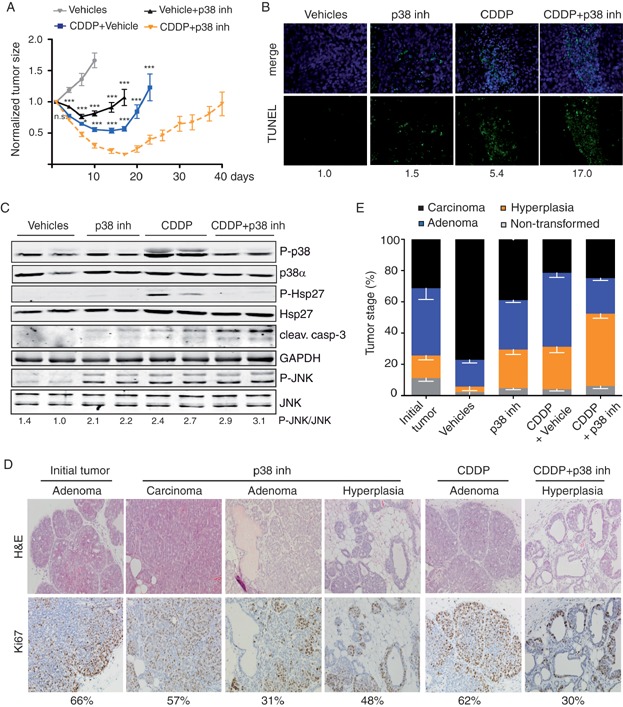
Cisplatin cooperates with p38 MAPK inhibition to reduce breast tumour size in mice Source data is available for this figure in the Supporting Information. MMTV-PyMT female mice with breast tumours of about 200 mm^3^ in volume were treated with a single-dose of cisplatin (CDDP) or vehicle followed by daily administration of PH-797804 (p38 inh) or vehicle for 15 days and tumour growth was monitored for up to 40 days. Tumour size was measured at the indicated times and normalized relative to the original size of each tumour when the treatment began. The graph compiles the results of three independent experiments, in which at least four mice were used per condition. ***p < 0.0001, *p < 0.05, ns = *p* > 0.025.Representative TUNEL staining of breast tumours collected at day 7. Average fluorescence intensities of the TUNEL images were quantified using FIJI, giving the value of 1 to the control (Vehicles).Breast tumours were collected at day 7 from two mice of each treatment and total lysates were analysed by immunoblotting with the indicated antibodies.H&E and Ki67 staining of the initial breast tumours and of tumours that relapsed back to 200 mm^3^ (original size) after the different treatments, as indicated in (A). Images shown are 20×. Quantifications of the Ki67 stainings are indicated.H&E stained sections of breast tumours obtained as in (D) were analysed in blinded fashion under the microscope and classified as carcinoma, adenoma, hyperplasia and normal tissue (non-transformed). Breast tumours treated only with vehicles and collected at day 10 (about 1.5× the original size) were also analysed to illustrate the normal progression of the untreated tumours to carcinomas.

To obtain *in vivo* evidence for the implication of p38 MAPK signalling in the response of cancer cells to cisplatin, we used the MMTV-PyMT mouse model of breast cancer. Female mice with PyMT-induced breast tumours were injected with a single dose of cisplatin (Evers et al, 2010) and then daily administered for 15 days either with the p38 MAPK inhibitor PH-797804 (Xing et al, 2009) or with vehicle, and tumour behaviour was monitored for up to 40 days. In agreement with our observations in human cancer cell lines, the combination of cisplatin treatment with p38 MAPK inhibition showed a clear additive effect and reduced the size of breast tumours in mice by more than 90% (Fig 7A). Analysis of these tumours 7 days after initiation of the treatment, showed that tumours treated with cisplatin and p38 MAPK inhibitor had increased levels of apoptosis as detected by TUNEL (Fig 7B) and by cleaved caspase 3 Western blotting (Fig 7C). Consistent with the results in human cancer cell lines, p38 MAPK inhibition also increased JNK activation in the mouse breast tumours, which was further enhanced in tumours of mice treated with the combined therapy (Fig 7C). Furthermore, we confirmed that cisplatin treatment induced p38 MAPK activation in mouse breast tumours (Fig 7C), and that treatment with the p38 MAPK inhibitor impaired activation of the p38 MAPK pathway (Supporting Information Fig S9A). Oxyblot analysis also showed increased levels of protein oxidation in tumour lysates from mice treated with the p38 MAPK inhibitor (Supporting Information Fig S9B). These results indicate that, in this model of breast cancer, the inhibition of p38 MAPK cooperates with cisplatin treatment to induce tumour cell death, which correlates with increased ROS and JNK pathway activation in the tumour cells. Immunohistochemical analysis confirmed reduced levels of cell proliferation, based on Ki67 staining, in the single treatments and to a greater extent in the double treatment at day 7 (Supporting Information Fig S9C). Similar results were observed at day 18 (Supporting Information Fig S10A) when the differences in size (Fig 7A) and in tumour grade (Supporting Information Fig S10B) where more evident.

We also monitored relapsing tumours and animals were sacrificed when breast tumours reached again the initial size (Fig 7A). Immunohistochemical analysis of initial tumours and the relapsed tumours obtained from mice that were subjected to the different treatments, confirmed that tumours from mice treated with cisplatin and the p38 MAPK inhibitor showed reduced cell proliferation (Fig 7D) and less advanced tumour grades (Fig 7E).

To determine if p38 MAPK inhibition could have additional effects on the response to cisplatin, relapsing tumours from mice initially treated with either cisplatin alone or in combination with the p38 MAPK inhibitor were treated again with another single dose of cisplatin. As previously reported (Rottenberg et al, 2007, 2012), cisplatin-treated tumours responded to the new treatment in exactly the same way as the first time. However, tumours that had been treated with the combined therapy behaved differently when treated again with cisplatin alone and tent to relapse faster back to the original size (Supporting Information Fig S11). These results indicate that p38 MAPK inhibition probably contributes to the delayed appearance of relapsing tumors observed in response to the combined therapy.

## DISCUSSION

It is well established that ROS play important biological roles in cell homeostasis, but several studies have also reported that high intracellular ROS levels are usually associated with apoptosis in cancer cells (Dolado et al, 2007; Wang & Yi, 2008). The JNK pathway is an important mediator of apoptosis induction in response to different chemotherapeutic agents, in particular cisplatin (Bae et al, 2006; Sanchez-Perez et al, 2000). We have found that the inhibition of p38 MAPK signalling in cancer cells suffices to increase ROS levels, which in turn stimulate JNK-mediated apoptosis in response to cisplatin. Previous reports have shown that the p38 MAPK and JNK pathways can crosstalk at several levels, mainly in non-transformed cells (Wagner & Nebreda, 2009), but this is the first time the interplay is shown to be mediated by ROS. We also provide evidence for the potential biological relevance of this mechanism in the response of cancer cells to chemotherapeutic treatments, extending previous reports on the implication of ROS in JNK-mediated apoptosis (Hou et al, 2008; Kamata et al, 2005).

ASK1 has been proposed as a key regulator of apoptosis in response to oxidative stress through the activation of MKK4 and MKK7 (Takeda et al, 2008). Nevertheless, upstream JNK activators are unlikely to be implicated in the JNK activation process that we describe here. Instead, our results indicate that the inhibition of JNK phosphatases by the accumulation of intracellular ROS may account for the hyperactivation of the JNK pathway upon p38 MAPK inhibition. It is known that ROS-induced oxidation of the catalytic cysteine can inhibit the activity of phosphatases (Karisch et al, 2011; Ostman et al, 2011). We have found no evidence in our experimental conditions that p38 MAPK inhibition affects the expression of the inducible nuclear phosphatase MKP1 (Keyse, 2008), which based on overexpression experiments was proposed to regulate cisplatin-induced JNK activation (Sanchez-Perez et al, 2000). However, by analysing cytoplasmic phosphatases that have been reported to target JNK phosphorylation (Dickinson & Keyse, 2006), it seems that different cysteine-based phosphatases are probably involved in the regulation of basal JNK activity depending on the cancer cell type.

We have also addressed how the inhibition of p38 MAPK increases ROS levels in cancer cells. Experiments using DPI rule out the implication of Nox enzymes in ROS accumulation induced by p38 MAPK inhibition. On the other hand, the use of rotenone suggests that electron transport through the mitochondrial respiratory chain may contribute to ROS generation after p38 MAPK inhibition. We have also identified two antioxidant genes, *GPX5* and *TXNDC2*, which are positively regulated by p38 MAPK signalling in cancer cells and control the basal JNK activity levels. Thus, p38α may negatively regulate ROS accumulation at different levels. Of note, p38α signalling has been reported to negatively regulate ROS accumulation in MEFs through the upregulation of catalase and superoxide dismutase (Gutierrez-Uzquiza et al, 2012), but we found no evidence for the regulation of these two enzymes by p38 MAPK in cancer cells. This indicates that p38α signalling can probably regulate ROS accumulation by different mechanisms in transformed cells. In fact, the different sensitivity of transformed and non-transformed cells to ROS has been proposed to be therapeutically useful (Mateescu et al, 2011; Raj et al, 2011; Shaw et al, 2011).

We have previously reported that p38α activation induces apoptosis in response to the expression of some oncogenes, whereas the JNK pathway does not seem to be involved in this process (Dolado et al, 2007). Contrary to the above scenario at the onset of cell transformation, we now show that enhanced JNK activity sensitizes cancer cells to apoptosis induced by chemotherapeutic drugs. This response seems to be conserved in different types of tumour cells, suggesting that it could be potentially exploited for cancer therapy in combination with oxidative stress-inducing agents (Engel & Evens, 2006). Consistent with this idea, expression of galectin 7 has been shown to upregulate ROS and JNK sensitizing urothelial cancer cells to cisplatin treatment (Matsui et al, 2007). Our results imply that whereas p38α plays a key role inducing apoptosis in response to ROS accumulation during tumour initiation (Dolado et al, 2007), this function seems to have been taken over by JNK in advanced tumour stages, where JNK hyperactivation sensitizes to apoptosis induced by chemotherapeutic agents. Thus, inhibition of p38α may sensitize tumour cells to these treatments.

Using a mouse model for breast cancer and a chemical inhibitor of p38 MAPK that is in clinical trials for inflammatory diseases (Goldstein et al, 2010; Hope et al, 2009), we confirmed *in vivo* that inhibition of p38 MAPK cooperates with cisplatin to kill tumour cells. Importantly, the breast tumours are not only smaller in the case of the combined therapy, but they seem to be in a less advanced stage based on histological analysis. Cell proliferation was also significantly reduced in these tumours, which could explain the delay in the re-appearance of the relapsing tumours.

Previous reports have shown that inhibition of p38 MAPK signalling may help chemotherapeutic agents to kill cancer cells. This is the case for myeloma cells treated with bortezomib (Navas et al, 2006), lymphoma cells treated with etoposide (Kurosu et al, 2005) or glioma cells treated with temozolomide (Demuth et al, 2007). Moreover, the downregulation of MAPKAPK2 (MK2), a downstream effector of p38α and p38β, has been shown to sensitize p53-deficient MEFs transformed with oncogenic Ras^G12V^ to doxorubicin or cisplatin treatment, both *in vitro* and upon subcutaneous implantation in nude mice (Reinhardt et al, 2007). In these cases, the enhanced cytotoxic effect observed upon inhibition of the p38 MAPK pathway has been associated with defective G2 cell cycle arrest. Recent work has also proposed that, in response to DNA damage, p38 MAPK signalling may play an important role regulating the expression of pro-survival proteins in the G2-arrested cells (Phong et al, 2010). We show that p38α and p38β inhibition results in ROS upregulation and JNK activation, sensitizing to cisplatin-induced tumour cell death both in human cancer cells and in a mouse model of breast cancer. It seems unlikely that defective cell cycle arrest would be associated with ROS upregulation in response to p38 MAPK inhibition, although we cannot rule out that other mechanisms might also contribute to the reduced tumour cell proliferation observed upon treatment with the p38 MAPK inhibitor. Moreover, the cooperative effect of p38 MAPK inhibition and cisplatin treatment is probably independent of p53, as we have observed this pro-apoptotic effect in cancer cells with different p53 status. Altogether, our results illustrate a new mechanism by which p38 MAPK contributes to tumour cell survival, and suggest that the combination of p38 MAPK inhibitors with chemotherapeutic agents should be considered for cancer therapy.

## MATERIALS AND METHODS

### Cell culture

Human breast (MCF7, MDA-MB-231) and colon (HT-29, SW620) cancer cells were maintained in Dulbecco's Modified Eagle's Medium (DMEM) supplemented with 10% heat-inactivated foetal bovine serum (FBS), 1% l-glutamine and 1% penicillin/streptomycin (all from GIBCO-Invitrogen). The HEK293-derived 293T cell line was used to produce shRNA-expressing viruses and it was maintained in the same media.

### Cell treatments and apoptosis assays

Cisplatin (Ferrer), H_2_O_2_ (Sigma), SB203580 (Axon MedChem), PH-797804 (Selleckchem) and SP600125 (Calbiochem) were added to the media of subconfluent cells from concentrated stocks: 0.5 mg/ml cisplatin, 8.8 M H_2_O_2_, 10 mM SB203580 (in DMSO), 2 mM PH-797804 (in DMSO) and 20 mM SP600125 (in DMSO).

The IC_50_ for treatments with cisplatin alone or in combination with the p38 MAPK inhibitor SB203580 were calculated for the HT29, SW620 and MCF7 cancer cell lines using an MTT assay (Cell Proliferation kit, Roche). Values were determined using GraphPad Prism introducing the doses in log(10) of the cisplatin concentrations (μM) and selecting a non-linear fit curve log with a symmetrical sigmoidal shape (Supporting Information Fig S12). Based on these data and the literature reporting the use of cisplatin at concentrations that range from 16 to 300 μM to treat cancer cell lines *in vitro* (Galan-Moya et al, 2011; Glorieux et al, 2011; Huang et al, 2003; Park et al, 2012; Ru et al, 2011), we decided to use 100 μM cisplatin to perform mechanistic studies at early time points.

For apoptosis assays, both floating and attached cells were collected and analysed using the Cell Death Detection kit ELISA PLUS (Roche Diagnostics) or the FITC Annexin V Apoptosis Detection kit I (BD Pharmingen), following manufacturer's instructions.

For antioxidant treatments, cells were pre-incubated for 1 h with a mixture of 5 mM *N*-acetyl cysteine (NAC, Sigma) and 5 mM reduced glutathione (GSH, Sigma) adjusted to pH 7.5. Antioxidants were maintained during further treatments of the cells.

### Cell cycle analysis

Cell-cycle distribution was analysed by flow cytometry. Cells were trypsinized and fixed in chilled 70% ethanol overnight. Cells were then incubated in PBS containing 0.2 mg/ml RNase A (Roche) and stained with 20 μg/ml propidium iodide (Sigma) for 30 min at 30°C. Flow-cytometry data was analysed with the FlowJo software.

### Immunoblotting

For preparation of cell lysates, subconfluent cell cultures were grown in 60 mm plates, washed twice in ice-cold PBS and immediately frozen on dry ice for −80°C storage. Cells were thawed on ice and directly scrapped in lysis buffer (50 mM Tris–HCl pH 7.5, 150 mM NaCl, 1% NP-40, 5 mM EDTA, 5 mM EGTA, 20 mM NaF, 0.1 mM sodium orthovanadate, 1 μM microcystin and the Protease Inhibitor Cocktail Set III from Merck Millipore). Breast tumours obtained from mice were mechanically disrupted in the same lysis buffer as above, but containing 1 mM DTT, with a Precellys apparatus (Bertin Technologies). The lysates were vortexed, incubated on ice for 10 min and centrifuged for 15 min at 16,000 × *g* and 4°C. Supernatants were quantified using the Protein Assay kit (Bio-Rad) with BSA as standard, and 20–50 μg of total protein were separated by SDS–PAGE. Proteins were transferred to nitrocellulose membranes (Protran, Schleicher & Schuell) using the Trans-well Blot® system (Bio-Rad). Membranes were blocked with 4% non-fat dry milk in PBS for 30 min at RT and then incubated with the following antibodies: phospho-c-Jun (9261S), phospho-MK2 (3007), phospho-p38 (9211), p38β (2339) and cleaved caspase 3 (9661) from Cell Signaling Technology; phospho-JNK (612541) and c-Jun (610327) from BD Biosciences; JNK (sc-571), HSP27 (sc-1049) and p38α (sc-535-G) from Santa Cruz Biotechnology; tubulin (T9026) and GAPDH (G8795) from Sigma; p85PARP (Promega, G7341), phospho-HSP27 (StressGen, SPA-523), catalase (Abgent, AM2118a) and SOD (Abcam, ab13533). All primary antibodies were diluted 1:500 in 5% BSA-PBS-Tween 0.1%, except for tubulin and GAPDH that were diluted 1:2000. After washing with PBS-Tween 0.1%, membranes were incubated with Alexa Fluor 680/800-conjugated secondary antibodies (Molecular Probes) diluted 1:5000 in 1% milk-PBS-Tween 0.1%, visualized and quantified using the Odyssey Infrared Imaging System (Li-Cor, Biosciences).

Oxidation of cellular proteins was measured using the OxyBlot™ Protein Oxidation Detection kit (Millipore) following the manufacturer's instructions. Standard immunoblotting was performed to detect oxidized protein residues.

### Clonogenic assays

Subconfluent cancer cells were incubated overnight for 1 h with cisplatin either 50 μM (HT-29, MCF7) or 10 μM (SW620). Then, 500 cells (HT-29, MCF7) or 1000 cells (SW620) were plated per well in six-well plates. Cells were allowed to grow until visible colonies were formed (about 2 weeks), and were stained with 0.5% crystal violet. Colonies were dissolved in methanol and the absorbance was read at 540 nm.

### siRNA-mediated knockdown

Human validated siRNA oligos were obtained from Ambion (Negative Control #1, 4390843; p38α-*MAPK14* #1, s3585; p38α-*MAPK14* #2, s3586; p38β-*MAPK11*, s11155; *DUSP8*, s4384; *DUSP10*, s22146), Dharmacon (*PTPN12*, L008064-00), Santa Cruz Biotechnology (*DUSP16*, sc-61052; GPX5, sc-62419; TXNDC2, sc-76575) and Sigma (synthesized for *PTPN1*: sense, 5′-UAGGUACAGAGACGUCAGUdTdT-3′ and antisense, 5′-ACUGACGUCUCUGUACCUAdTdT-3′; JNK1/2: sense, 5′-AAAGAAUGUCCUACCUUC UdTdTdTdT-3′ and antisense, 5′-AGAAGGUAGGACAUUCUUUdTdTdTdT-3′). MCF-7 cells were transfected with 50 nM siRNA using DharmaFECT-1 buffer (Dharmacon) and collected 48 h later. Protein lysates were analysed by immunoblotting and RNA was purified to confirm downregulation of the target genes by qRT-PCR using the primers indicated in Supporting Information Table S2.

### Lentiviral infections

JNK1 and JNK2 shRNAs were provided by Erwin Wagner (CNIO, Madrid, Spain). COX-2 and p38α shRNAs were obtained from the human MISSION™ shRNA library (Sigma-Aldrich). Lentivirus were produced in 293T cells by transient co-transfection of the MISSION® Lentiviral Packaging Mix (Sigma) with shRNA-encoding DNA. Culture supernatants were collected 72 h post-transfection, filtered (0.45 mm filter, PVDF, Millipore), and supplemented with 8 μg/ml polybrene (Sigma). Subconfluent cells were infected with the polybrene-supplemented supernatant and 48 h post-infection were selected with 1 μg/ml puromycin for 1 week.

**The paper explained**

PROBLEM:

There is a need to design therapies that specifically target tumour cells in order to minimize collateral effects. Recent studies support that the high levels of ROS present in tumour cells could be therapeutically used to target various cancers. The p38 MAPK pathway is known to function both as a sensor and a regulator of cellular ROS levels. Moreover, p38 MAPK has been proposed to contribute to the proliferation and survival of different types of tumour cells and p38 MAPK overactivation has been correlated with poor prognosis in some cancer patients. However, there is little *in vivo* evidence supporting that p38 MAPK inhibition could potentiate the effect of chemotherapy.

RESULTS:

We show that inhibition of p38 MAPK cooperates with the chemotherapeutic drug cisplatin to induce apoptosis in different types of human tumour cells. The underlying mechanism relays on the upregulation of ROS levels upon p38 MAPK inhibition. This leads to hyperactivation of the JNK pathway, which is responsible for triggering tumour cell death. Importantly, we show that the combined therapy reduces the size and malignancy of tumours *in vivo* using a genetic mouse model of breast cancer.

IMPACT:

Our results highlight the potential therapeutic interest of combining p38 MAPK chemical inhibitors, which are currently in clinical trials, with cancer chemotherapeutic drugs such as cisplatin. In addition, the increased ROS levels induced by impairing p38 MAPK signalling suggest that inhibitors of this pathway might sensitize cancer cells to the effect of other anti-tumoural treatments.

### Determination of intracellular ROS levels

Subconfluent cells were incubated for 30 min at 37°C with 10 μM DCFDA (2′,7′-dichlorodihydrofluorescein diacetate, Sigma) and then were trypsinized and resuspended in 1 ml of PBS with 1.4 μg/ml aprotinin (Sigma). After counter-staining using propidium iodide, cells were analysed by flow cytometry using the FlowJo software.

### Gene expression arrays

The relative expression levels of 84 genes involved in ROS regulation was evaluated using the Human Oxidative Stress and Antioxidant Defense RT^2^ Profiler™ PCR array (SABiosciences) according to the manufacturer's instructions. Briefly, DNase-treated total RNA from MCF7 cells, either untreated or treated with 10 μM SB203580, was purified with the RNeasy minikit (Qiagen). cDNAs were generated by reverse transcription from 4 µg of total RNA from each sample using the RT^2^ First Strand Kit, then combined with the RT^2^ qPCR Master Mix and added to lyophilized primer pairs in the 96 well plate arrays. Thermal cycling was performed in a CFX96™ Real Time System (BioRad) and relative gene expression levels were calculated using the ΔΔ*C*_t_ method with normalization to the average expression level of three common genes (*GAPDH*, *HPRT1* and *RPL13A*). Selected genes were validated by qRT-PCR using the primers indicated in Supporting Information Table S2.

### Phosphatase assays

Plasmids to express GST-JNK1 and GST-MKK7 proteins were kindly provided by Carme Caelles (University of Barcelona, Spain) and Roger Davis (University of Massachusetts Medical School, USA), respectively. Proteins were expressed in *E. coli* BL21 (DE3). Recombinant GST-JNK1 (120 μg) was phosphorylated *in vitro* with GST-MKK7 (12 μg) and [γ-^32^P] ATP (20 μCi) in a buffer containing 50 mM Tris pH 7.5, 10 mM MgCl_2_ and 1 mM DTT. Phosphorylated GST-JNK1 was bound to GST beads (Glutathione Sepharose 4B, GE Healthcare) and resuspended in phosphatase buffer (50 mM Tris pH 7.5, 250 mM NaCl, 1% Triton X100, 1 mM PMSF and 10 μg/ml of aprotinin, leupeptin and pepstatin). About 0.5 μg of GST-JNK1 were incubated in a total volume of 52 μl with 100 μg of total lysate prepared from cells that had been pre-treated or not with 10 μM SB203580 for 6 h. As a control, the assay was also performed in the presence of the phosphatase inhibitors NaF (20 mM), sodium orthovanadate (0.1 mM) and microcystin (2 μM). Samples were taken at the indicated time points and were analysed by SDS–PAGE and autoradiography (PhosphorImager).

Generic phosphatase activity was measured using commercial Tyr and Ser/Thr Phosphatase Assay kits (Promega) following the manufacturer's instructions. Briefly, reactions were performed in 50 μl of volume containing 5 μg of phospho-peptide and 750 μg of cell lysate prepared in 50 mM Tris pH 7.5, 250 mM NaCl and 0.1 mM DTT buffer. After incubation at 30°C for the indicated times, the reaction was stopped by addition of 50 μl of molybdate dye and absorbance was measured at 630 nm.

### PyMT mice

MMTV-PyMT mice (Guy et al, 1992) were provided by William Muller (McGill University, Canada). Female mice expressing MMTV-PyMT spontaneously develop breast tumours with 100% penetrance. When breast tumours reached a size of around 200 mm^3^ (*V* = (*π* × *l*)×*w*^2^), mice were given a single injection of 6 mg/kg cisplatin (Accord, 683046-8, stock 1 mg/ml) or vehicle (physiological saline) in the tail vein. The p38 MAPK inhibitor PH-797804 (Selleckchem) (Hope et al, 2009) was prepared at 1 mg/ml in PBS with 0.5% Methyl cellulose (Sigma) and 0.025% Tween 20 (Sigma) and administered daily to the mice by oral gavage (10 mg/kg). Groups of at least four mice (each one of them developing between two and five tumours) were used per condition in every experiment. Mice were housed according to national and EU regulations, and protocols were approved by the Ethics committee of our Institute.

### Histology and immunohistochemistry

For histological analysis, formalin-fixed, paraffin-embedded breast tumours were stained with haematoxylin and eosin (H&E) and analysed in blinded fashion by two independent observers under the microscope. For each mouse, at least 10 high power fields (200×) from two tissue slides of two different tumours were analysed. The malignancy was determined based on the microscopic appearance of the tissues (degree of filled tubules, rate of cell division, cell size and uniformity) and classified into four stages: normal tissue, hyperplasia, adenocarcinoma and carcinoma. Hyperplasic tissues resemble normal cells, arranged around tubules, well differentiated and with a low proliferation rate. Adenomas show moderately differentiated cells, which still retain the glandular-related tissue cytology and architecture, with clear tumour margins. Carcinomas show very abnormal and poorly differentiated cells with no significant resemblance to the corresponding parent cells and tissue.

To determine cell proliferation, formalin-fixed, paraffin-embedded breast tumours were stained with an antibody against Ki67 (Novacastra), using as secondary antibody a HRP conjugated anti-rabbit (ImmunoLogic). Signal was visualized with DAB (3,3-diaminobenzidine) using H&E as a counter staining. For apoptosis detection by TUNEL, we used the “*In situ* Cell Death Detection Kit, Fluorescein” (Roche), following the manufacturer's instructions.

### Statistical analysis

For ROS measurements, data were transformed logarithmically to satisfy the Gaussian criterion and *p*-values were calculated using an unpaired two-tailed Student's *t*-test at a significance level of *α* = 0.05, under the null hypothesis that the mean is equal to zero. For other assays, *p*-values were calculated using an unpaired two-tailed Student's *t*-test at a significance level of *α* = 0.05. For mouse tumour sizes, we compared each treatment (p38 inh or CDDP) with the double therapy (CDDP + p38 inh) with the non-parametric Kruskall–Wallis test, followed by Mann–Whitney *U*-test pair-wise comparisons and adjusted *p*-value by the Bonferroni correction at a significance level of *α* = 0.025.

## Supplementary material

As a service to our authors and readers, this journal provides supporting information supplied by the authors. Such materials are peer reviewed and may be re-organized for online delivery, but are not copy-edited or typeset. Technical support issues arising from supporting information (other than missing files) should be addressed to the authors.

**Figure S1.** A. MCF7 cells were treated with increasing concentrations of SB203580 (SB, 1–10 µM) for 6 h. Total cell lysates were analysed by immunoblotting with the indicated antibodies. B. HT-29 and SW620 cells were pre-incubated for 2 h with PH-797804 (PH, 2 µM) and then treated with cisplatin (CDDP, 100 µM) for 24 h. Cell death was measured with propidium iodide (PI) and Annexin V staining. The percentages of apoptotic cells are indicated. C. SW620 cells were incubated overnight with PH-797804 (PH, 2 µM) and then treated for 8 h with cisplatin (CDDP, 100 µM). Total cell lysates were analysed by immunoblotting with the indicated antibodies.

**Figure S2.** A. MCF7 cells were transfected with siRNAs (50 nM) either control or against p38, p38β or both together. After 48 h, cells were treated for 8 h with cisplatin (CDDP, 100 µM). Total lysates were analysed by immunoblotting with the indicated antibodies. The upper blot was incubated first with the p38β antibody and then with the p38α antibody. B. HT-29 cells were transfected with siRNAs (50 nM) either control or against JNK1/2. After 48 h, cells were incubated for 2 h in the presence or absence of PH-797804 (PH) and then treated for 24 h with cisplatin (CDDP, 100 µM). Cell death was quantified by staining with propidium iodide (PI) and Annexin V. Total cell lysates were analysed by immunoblotting with the indicated antibodies (right panel). C. HT-29 cells were infected with lentiviruses expressing shRNAs against JNK1 or JNK2 or a non-targeting control. Pools of cells were incubated overnight in the presence or absence of SB203580 (SB, 10 µM) and then treated for 8 h with cisplatin (CDDP, 100 µM). Total cell lysates were analysed by immunoblotting with the indicated antibodies.

**Figure S3.** A. HT-29 and MCF7 cells were incubated for the indicated times with SB203580 (SB, 10 µM), overnight with DMSO, or 10 min with H2O2 (5 mM). ROS levels were measured with DCFDA by flow cytometry. B. MCF7 cells were pre-treated for 1 h with a mixture of NAC and GSH antioxidants (AOX) and then incubated overnight with SB203580 (SB, 10 µM), or were treated with 5 mM H2O2 for 1 h, as indicated. Cell lysates were analysed by immunoblotting using Oxyblot and the total protein oxidation signal per lane was quantified using tubulin as a reference. C. MCF7 cells were pre-treated for 1 h with antioxidants (AOX) as in (B), followed by overnight incubation with PH-797804 (PH, 2 µM) and then treated with cisplatin (CDDP, 100 µM) for 8 h. Total cell lysates were analysed by immunoblotting with the indicated antibodies.

**Figure S4. A.** Cells were treated for 1 h with a mixture of NAC and GSH antioxidants (AOX) or incubated overnight with SB203580 (SB, 10 µM) and then plated for clonogenic assays. Colonies were dissolved in methanol and the absorbance reads (540 nm) are represented in the bar diagrams. B. Cells were incubated for 48 h either with two different siRNAs against p38α (#1 and #2) or with a scrambled siRNA (siCtrl) as a control. Total cell lysates were analysed by immunoblotting with the indicated antibodies (upper blot). For clonogenic assays, siRNA-treated cells were treated for 1 h with AOX as in (A) followed by 1 h incubation with cisplatin (CDDP) and then plated. Colonies were dissolved in methanol and the absorbance reads (540 nm) are represented in the bar diagrams.

**Figure S5.** MDA-MB-231 cells were incubated for the indicated times with SB203580 (SB, 10 mM), overnight with DMSO or 10 min with H_2_O_2_ (5 mM). ROS levels were measured with DCFDA by flow cytometry.

**Figure S6.** MCF7, MDA-MB-231 and SW620 cancer cell lines were treated overnight with SB203580 (SB, 10 µM). Total cell lysates were analysed by immunoblotting with the indicated antibodies. CAT, catalase; SOD, superoxide dismutase.

**Figure S7.** The indicated cancer cell lines were transfected with siRNAs (50 nM) against PTPN1, PTPN12, DUSP16, DUSP8 and DUSP10, or with a scramble control, and 48 h later total cell lysates were analysed by immunoblotting with the indicated antibodies. As a positive control, cells were incubated overnight with SB203580 (SB, 10 µM) before immunoblotting.

**Figure S8.** A. MCF7 cells were incubated with the indicated concentrations of ASK1 inhibitor (ASK1 inh) overnight and then irradiated with UV (50 J/m^2^) followed by 30 min incubation at 37°C. Total cell lysates were analysed by immunoblotting with the indicated antibodies. B. MCF7 cells were incubated overnight with SB203580 (p38 inh, 10 µM) and/or ASK1 inhibitor (10 µM) followed by cisplatin (CDDP, 100 µM) for 8 h. Total cell lysates were analysed by immunoblotting with the indicated antibodies.

**Figure S9.** A. Breast tumours obtained from different treatments were incubated, immediatly after resection, for 15 min in a solution of NaCl (300 mM) and then snap frozen. Total tissue lysates were analysed by immunoblotting with the indicated antibodies. B. Total lysates from breast tumours at day 7 were analysed by immunoblotting using Oxyblot. The total protein oxidation signal per lane was quantified with tubulin as a reference. Results were confirmed using three mice per condition. C. H&E and Ki67 staining of breast tumours analysed at day 7. Quantifications of Ki67 staining are indicated. Images are 20×.

**Figure S10.** A. H&E and Ki67 staining of breast tumours collected at day 18. Images shown are 20×. Quantifications of Ki67 staining are indicated. B. H&E stained sections of breast tumours collected at day 18 were analysed under the microscope in blinded fashion. Samples were classified as carcinoma, adenoma, hyperplasia and normal tissue (non-transformed).

**Figure S11.** MMTV-PyMT female mice with breast tumours of about 200 mm^3^ in volume were treated with a single-dose of cisplatin (CDDP) followed by daily administration of PH-797804 (PH, 10 mg/kg) or vehicle for 15 days and tumour growth was monitored until tumors reached again 200 mm^3^. Then, a second injection of CDDP was administered to the mice and tumour growth was monitored for up to 70 days. Tumour size was measured at the indicated times and normalized relative to the original size of each tumour when the treatment began. The graph compiles the results of three independent experiments, in which at least four mice per condition were used.

**Figure S12.** MCF7, SW620 and HT-29 cells were treated with increasing concentrations of cisplatin (CDDP) with or without SB203580 (SB, 10 µM) for 24 h. Cell viability was measured using the MTT assay. The percentages of viable cells relative to non-treated cells were calculated.

**Table S1.** Antioxidant enzyme-encoding genes regulated by p38 MAPK in cancer cells.

**Table S2.** Sequences of primers used for RT-PCR analysis.
