# Supplementary material for: Inhibition of p38 MAPK sensitizes tumour cells to cisplatin-induced apoptosis mediated by reactive oxygen species and JNK
Source: EMBO Mol Med. 2013 Sep 24;5(11):1759–74. doi: 10.1002/emmm.201302732 (PMC3840490; doi:10.1002/emmm.201302732)
Supplement: Supplementary file 1 [file emmm0005-1759-SD1.pdf]

EMBO Molecular Medicine

Inhibition of p38 MAPK sensitizes tumor cells to cisplatin-induced apoptosis mediated by reactive oxygen species and JNK

Lorena Pereira, Ana Igea, Begoña Canovas, Ignacio Dolado, and Angel R. Nebreda

Corresponding author: Angel Nebreda, IRB Barcelona

Review timeline:

Submission date:	08 March 2013
Editorial Decision:	15 March 2013
Appeal received:	18 March 2013
Editorial Decision:	10 April 2013
Revision received:	01 August 2013
Editorial Decision:	19 August 2013
Revision received:	26 August 2013
Accepted:	27 August 2013

Transaction Report:

(Note: With the exception of the correction of typographical or spelling errors that could be a source of ambiguity, letters and reports are not edited. The original formatting of letters and referee reports may not be reflected in this compilation.)

Editor: Céline Carret

1st Editorial Decision 15 March 2013

Thank you for the submission of your manuscript "Inhibition of p38 MAPK sensitizes tumor cells to cisplatin-induced apoptosis mediated by reactive oxygen species and JNK".

I have now had the opportunity to carefully read your paper and the related literature and I have also discussed it with my colleagues and an Editorial Advisory Board member. I am afraid that we decided that the manuscript is not well suited for publication in EMBO Molecular Medicine and have therefore decided not to proceed with peer review.

We appreciate that p38 inhibitor used in vitro and in vivo (in transgenic breast cancer mouse model) in combination with cisplatin synergistically affects tumor growth. Mechanistically, inhibition of p38a results in elevated ROS levels that block phosphatases and activate the JNK pathway to re-sensitize cells to cisplatin-induced apoptosis.

However, you already showed that p38a MAPK was a sensor of ROS in tumorigenesis. Therefore, we feel that the current manuscript constitutes a clinical demonstration of your previously published findings. While we understand that never before was ROS implicated in mediating an interplay between p38 and JNK, JNK and ROS axis on one hand and ROS and p38 on another hand have been reported before in cancer signaling. As such, while we do find the study well-done and conclusive, and do appreciate the clinical aspect of the work, we are nevertheless not persuaded that it is of sufficient conceptual advance and general interest for a broad translational journal like ours.

As this opinion was shared by the Editorial Advisory Board member we consulted with, I am afraid that I cannot offer further proceedings in EMBO Molecular Medicine.

I am sorry that I could not bring better news this time.

Appeal received 18 March 2013

Thank you for your message. We are obviously disappointed that you found our paper unsuitable for EMBO Mol Med. However, after reading your comments we have noticed that your decision might have been based on a misinterpretation of our results.

We would like to call your attention to a couple of important points:

1. Our manuscript does not provide a clinical demonstration of our previously published findings; in fact, this is a totally different story. Our previous work showed that p38a acts as a tumor suppressor by sensing ROS accumulation (ROS activates p38a) and inducing apoptosis. This is one of the mechanisms that cells can use to avoid oncogene-induced transformation (Dolado et al, 2007). On the contrary, we now show that p38a regulates production of ROS (not the other way around), which plays an important role in the response of cancer cells to chemotherapeutic drugs. Thus, the new findings show that p38a plays opposite roles depending on whether cells are undergoing transformation or are fully transformed, probably due to the rewiring of this signaling pathway. In the first case, p38a induces apoptosis (by sensing of ROS), in the second case p38a inhibits apoptosis (by regulating ROS production).

2. A quick look at PubMed using ROS, JNK and p38 as keywords certainly identifies a number of published reports, but careful examination shows that these reports mainly describe the activation of both p38a and JNK by ROS, which is not the point of our story. We propose a well-defined mechanism whereby downregulation of p38a induces the accumulation of ROS sensitizing cancer cells to apoptosis mediated by JNK.

We mention two studies performed in primary fibroblasts and pancreatic b-cells, respectively, where ROS production activates JNK by inhibition of phosphatases (Hou et al, 2008; Kamata et al, 2005). Such a mechanism based on the regulation of endogenous phosphatases has not been described in cancer cells.

We also cite two papers that link p38a with ROS production. The study of Gutierrez-Uzquiza et al (2012) was performed in primary fibroblasts and proposes a different mechanism from what we describe. Mateescu et al (2011) mentions only a correlation in cancer cells based on gene expression signatures but no mechanism is proposed. To our knowledge, we describe the first mechanism by which p38a regulates ROS production in epithelial cancer cells.

Independently of the new mechanistic aspects, the validation of our results using a mouse model of cancer and the availability of chemical inhibitors for p38a provides a pre-clinical perspective to the study, which is what we thought the manuscript would appeal to EMBO Mol Med.

We understand that as an Editor you can choose what to publish, as it should be. But it sends the wrong message that a journal of the standing of EMBO Mol Med could appear to be taking decisions based on erroneous scientific arguments.

2nd Editorial Decision 10 April 2013

Thank you for the submission of your manuscript to EMBO Molecular Medicine. We have now heard back from the three referees whom we asked to evaluate your manuscript. Although the referees find the study to be of potential interest, they also raise a significant number of issues that have to be addressed in a major revision of your work.

As you will see from the reports below, all three referees are concerned about the conclusiveness of the study and suggest a number of experiments to improve and strengthen the data. While we do find all issues raised of importance, I would like to particularly point out that the cisplatin doses in vitro should be lowered to more realistic value and control data provided (IC50s assessed for all drug/combination used, combination indexes determined). In vivo, P38 expression should be determined in tumors following drug treatment and PH797804 inhibitor showed to reverse p38 activation in order to increase the clinical relevance of the experiments. In addition, and as suggested by referees #1 and #2, knockdown of p38beta should be done to confirm inhibitors data.

Given these evaluations, I would like to give you the opportunity to revise your manuscript, with the understanding that the referees' concerns must be fully addressed and that acceptance of the manuscript would entail a second round of review.

Please note that it is EMBO Molecular Medicine policy to allow a single round of revision in order to avoid the delayed publication of research findings. Consequently, acceptance or rejection of the manuscript will depend on the completeness of your responses included in the next version of the manuscript.

EMBO Molecular Medicine has a "scooping protection" policy, whereby similar findings that are published by others during review or revision are not a criterion for rejection. Should you decide to submit a revised version, I do ask that you get in touch after three months if you have not completed it, to update us on the status.

Please also contact us as soon as possible if similar work is published elsewhere. If other work is published we may not be able to extend the revision period beyond three months.

I look forward to seeing a revised form of your manuscript as soon as possible.

***** Reviewer's comments *****

Referee #1 (Comments on Novelty/Model System):

My major criticism of the work is the focus on p38alpha. Both inhibitors used are not isoform specific and will also inhibit p38beta. I believe a p38beta knockdown would complete the story and strengthen the paper considerably.

Referee #1 (Remarks):

The manuscript by Pereira et al. describes a synergistic effect of cisplatin and p38alpha/beta inhibition on tumour cell death. They use the p38alpha/beta inhibitors SB203580 and PH-797804 and siRNA for p38alpha. In order to make a strong case, the authors would have to include a p38beta knockdown as well.

Abstract: As SB203580 inhibits both, p38alpha and p38beta, the abstract should at this stage not talk about p38alpha only.
p. 5, line 7: PARP needs to be defined.
p. 9: PH-797804 treatment again is described as p38alpha inhibition, but that PH-797804 is not isoform-specific, even though it is about 10 times more active against p38alpha than p38beta.
p. 13, line 9: N-acetyl cysteine: italicise "N"
p. 13, line 5 from bottom: "Schleicher & Schnell" should read "Schleicher & Schuell". The dilutions of the primary and secondary antibodies used should be clearly stated.
p. 17, Statistical analysis and figures 1-3, 6: CDDP is not defined.
Figure legends: All abbreviations used in the figures need to be explained in the accompanying legends.
Supporting information S2 and S4: H2O2 should read H_2O_2.

Referee #2 (Comments on Novelty/Model System):

point 2: The authors mention in the introduction that it is already known that cisplatin resistance is linked to increased MAPK signalling. Overall it is somewhat unclear to me what is really new to justify publication in EMM.

point 4: is p38 really induced in the in vivo model?

Referee #2 (Remarks):

In this manuscript the authors claim that p38a inhibition sensitizes tumor cells to cisplatin-induced apoptosis due to increased ROS levels and JNK pathway activation. For this purpose the authors perform experiments in 3 cell lines (HT-29, SW620, MCF7) and for in vivo validation they use the PyMT mouse model for breast cancer.

Overall, I do not find the data conclusive, and I have the following major concerns:

- In all of the in vitro experiments a cisplatin concentration of 100microM was used. This is totally unrealistic. Such concentrations will never be achieved in real tumors, and therefore all of this work is not informative. A concentration of 10microM cisplatin may be reached temporarily in vivo.
- I miss more direct evidence that p38 is induced after cisplatin treatment. HSP27 phosphorylation is more indirect. Is p38 still induced when 10microM of cisplatin are used?
- For the RNAi experiments (Fig. 2 and Fig. 3) I miss deconvolution. Can the effect be reproduced with at least 2 independent shRNAs/siRNAs?
- What is the effect on clonogenic assays when independent siRNAs against p38 are used?
- In vivo a different p38 inhibitor is used than in vitro. Why?
- In vitro the authors genetically inhibit p38 as additional control to chemical inhibition. I miss an experiment in which p38 is also genetically inactivated in vivo.
- Is p38 activated in the in vivo model after cisplatin treatment?
- Do the authors manage to inhibit p38 successfully in vivo using PH-797804?
- Fig. 6A: The additional effect of p38 inhibition over cisplatin may be very small. Was the response monitored over a longer time than just 18 days? When do tumors relapse back to 200mm3? Why was the vehicle not added to cisplatin?
- I miss quantification of the data presented in Fig. 6C and D. Moreover, The TUNEL pictures of 6C are hardly visible in the pdf copy (maybe due to low resolution pdf?). I think they require a higher quality picture.
- Figure 6F: This is not useful, since this is apparently done on day 18, when tumors have shrunk after combination therapy. Small residual tumor nests may be miss-interpreted as adenoma. What about the tumor when it relapsed back to 200mm3 ?
- On page 9 the authors even state that "a better prognosis could be expected in the event of a possible relapse after therapy termination." I do not find this serious. Usually relapsing tumors are more resistant. If the authors consider that there may be a better prognosis of relapsing tumors, why did they not test it?
- The authors mention in the introduction that it is already known that cisplatin resistance is linked to increased MAPK signalling. Overall it is somewhat unclear to me what is really new to justify publication in EMM.

Referee #3 (Remarks):

This manuscript demonstrates the potential clinical significance of cross talk between the p38/RK and JNK MAP Kinase pathways in the response of tumour cells to CDDP. The authors show that p38 inhibition drives the production of ROS to inactivate a JNK phosphatase, thereby activation JNK to drive apoptosis. Such mechanism has been proposed in the literature before but based on very poor or flimsy data. This is a convincing study that also demonstrates the potential clinical significance in a mouse model of cancer.

There are however some points that need to be addressed before publication

1. Figure 1B. This figure needs the data for 'SB alone' to be included.
2. Figure 1D needs the 'control' and 'SB alone' data included so that the reader can clearly see the degree of synergy or additivity between the treatment regimens.
3. Why does the JNK inhibitor SP600125 inhibit JNK phosphorylation in Figure 1F? This drug is an inhibitor of JNK not MKK4 or MKK7
4. The ability of JNK inhibition (SP600125 or shRNA) to overcome the cell death observed with CDDP+SB is only shown using PARP cleavage (Figure 1F and 2D), a qualitative assay. This is a very important part of the study and needs to be demonstrated with robust quantitative assays (DNA oligonucleosomes, sub-G1 or Annexin-V) and appropriate statistics.
5. The ability of ROS scavengers to protect cells from CDDP+SB is also only shown by PARP cleavage (Figure 3D and 3F) and needs to be confirmed with quantitative assays.
6. It is very interesting that CDDP and SB cooperate to activate JNK and promote cell death in SW620 cells as these cells have a mutation in MKK4 that generates a truncated non-functional protein. This suggests that JNK activation is exclusively via MKK7 in these cells? A MKK7 siRNA experiment here would add significant mechanistic insight.
7. Figure 5C. The blots indicate that DUSP16 knockdown also increases JNK phosphorylation - especially the p54 forms. Might this explain why DUSP8 is not the key regulator in other cells (Fig S6)?

Minor points

1. Penultimate line of page 7. Oxidation spelt incorrectly ('inactivation by oxidation').

1st Revision - authors' response 01 August 2013

Revised manuscript EMM-2013-02732. Response to Reviewers.

Referee #1 (Comments on Novelty/Model System):

My major criticism of the work is the focus on p38alpha. Both inhibitors used are not isoform specific and will also inhibit p38beta. I believe a p38beta knockdown would complete the story and strengthen the paper considerably.

This is a good point that was also mentioned by Referee #2. In the revised manuscript we have addressed this issue by comparing the effect of down-regulating either p38a or p38b in cisplatin-induced cell death (New Fig 2C, 2D and Supporting Information S2A). We observed that p38b knockdown promotes cisplatin-induced cell death, but the effect was smaller than with p38a knockdown, indicating that p38a plays a major role in the response to cisplatin. We also found that the combined knockdown of p38a and p38b resulted in higher levels of cell death than the individual knockdowns further supporting that both are implicated. We have rewritten the text.

Referee #1 (Remarks):

The manuscript by Pereira et al. describes a synergistic effect of cisplatin and p38alpha/beta inhibition on tumour cell death. They use the p38alpha/beta inhibitors SB203580 and PH-797804 and siRNA for p38alpha. In order to make a strong case, the authors would have to include a p38beta knockdown as well.

Abstract: As SB203580 inhibits both, p38alpha and p38beta, the abstract should at this stage not talk about p38alpha only.
p. 5, line 7: PARP needs to be defined.
p. 9: PH-797804 treatment again is described as p38alpha inhibition, but that PH-797804 is not isoform-specific, even though it is about 10 times more active against p38alpha than p38beta.
p. 13, line 9: N-acetyl cysteine: italicise "N"
p. 13, line 5 from bottom: "Schleicher & Schnell" should read "Schleicher & Schuell". The dilutions of the primary and secondary antibodies used should be clearly stated.
p. 17, Statistical analysis and figures 1-3, 6: CDDP is not defined.

Figure legends: All abbreviations used in the figures need to be explained in the accompanying legends.
Supporting information S2 and S4: H2O2 should read H_2O_2.

All the suggested corrections have been introduced in the main text and figures.

Referee #2 (Comments on Novelty/Model System):

point 2: The authors mention in the introduction that it is already known that cisplatin resistance is linked to increased MAPK signaling. Overall it is somewhat unclear to me what is really new to justify publication in EMM.

The sentence in the Introduction was misleading and we apologize for not being clearer. In the first paper that we cited (Galan-Moya et al, 2011), cell lines derived from non-small cell lung cancer or head and neck carcinomas are used to show that sensitivity to cisplatin is associated with activation of p38 MAPK. Intriguingly, this paper also shows that cancer cell lines with high basal levels of p38 MAPK activity, which correlate with high expression of the MKK3 upstream activator, tend to be more resistant to cisplatin. The authors propose that resistance correlates with the inability to further activate p38 MAPK in response to cisplatin. This manuscript provides no information on the mechanism by which p38 MAPK signaling could impinge on cisplatin sensitivity or resistance. We have now clarified this in the revised manuscript. In the second paper (Chung et al, 2012), knockdown of galectin-1 in lung cancer cells lines is reported to enhance sensitivity to cisplatin, which correlates with down-regulation of ERK, p38 MAPK and NFkB as well as attenuated expression of COX-2. After careful re-evaluation of this paper, we have not found any experiment to confirm the implication of p38 MAPK in the reported effects and we have therefore removed the reference from the revised manuscript.

Our work provides evidence for a new mechanism that explains why inhibition of p38 MAPK sensitizes cancer cells to apoptosis. We show that p38 MAPK maintains low basal levels of ROS and phosphorylated JNK in cancer cells. As a consequence, inhibition of p38 MAPK enhances the pool of phosphorylated JNK, which in turn induces apoptosis of the cancer cell more efficiently after cisplatin treatment. This applies to cancer cells that have different levels of basal p38 MAPK activity. Moreover, we provide to our knowledge the first *in vivo* evidence that inhibition of p38 MAPK sensitizes tumor cells to cisplatin-induced apoptosis.

point 4: is p38 really induced in the in vivo model?

We agree that this important point was missing in our original submission. We now show that cisplatin treatment induces the phosphorylation of p38 MAPK in the activation sites, which is required for and correlates with kinase activity (New Fig 7C).

Referee #2 (Remarks):

In this manuscript the authors claim that p38a inhibition sensitizes tumor cells to cisplatin-induced apoptosis due to increased ROS levels and JNK pathway activation. For this purpose the authors perform experiments in 3 cell lines (HT-29, SW620, MCF7) and for in vivo validation they use the PyMT mouse model for breast cancer. Overall, I do not find the data conclusive, and I have the following major concerns:

- In all of the in vitro experiments a cisplatin concentration of 100microM was used. This is totally unrealistic. Such concentrations will never be achieved in real tumors, and therefore all of this work is not informative. A concentration of 10microM cisplatin may be reached temporarily in vivo.

We agree with the Referee that the cisplatin dose we used might seem high, but the purpose of this study was to elucidate the triggering mechanism of the phenotype observed. To be able to perform the mechanistic studies in the different cancer cell lines independently of their susceptibility, we decided to use a dose of 100 mM and an early time point (8 h)
Following the Referee's comments, we have now used the MTT assay to determine the IC_{50} of MCF7, SW620 and HT-29 cells after 24 h of cisplatin treatment. The values obtained were 47, 73

and 370 mM, respectively (Supporting Information Figure S12), which were not so different from the dose used in our experiments. The combination indexes with SB203580 were in all the cases lower (20 µM for MCF7, 30 µM for SW620 and 309 µM for HT-29).
We have also investigated the effects of 10 mM cisplatin and observed that significant cell death was only induced after 4 days of in vitro treatment but not at shorter time points. Importantly, cell death induced by 10 mM cisplatin was enhanced upon incubation with two different p38 MAPK inhibitors (see below).

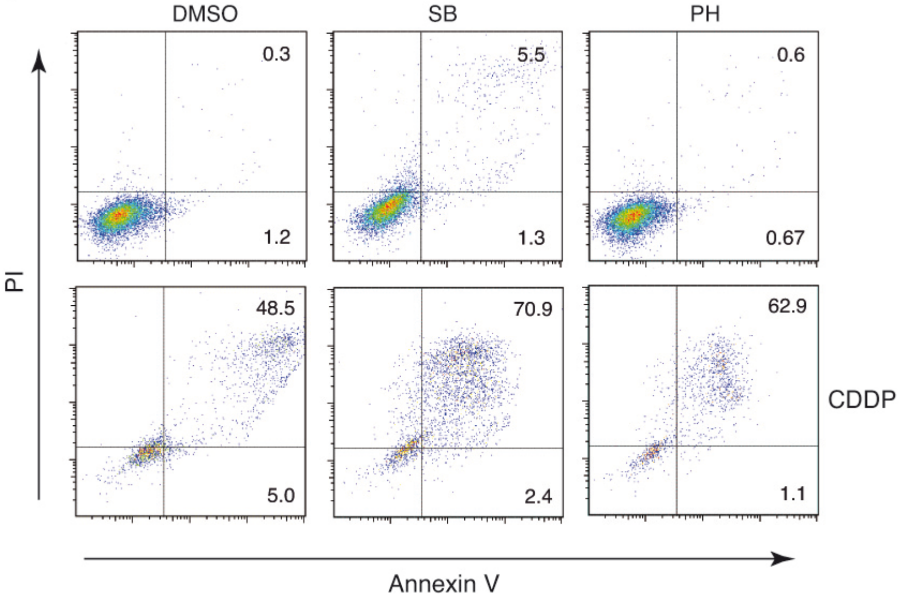

SW620 cells were pre-incubated for 2 h with SB203580 (SB, 10 µM) or PH-797804 (PH, 2 µM) and then treated with cisplatin (CDDP, 10 µM) for 96 h. Cell death was measured by propidium iodide (PI) and Annexin V staining. The percentages of apoptotic cells are indicated.

In our opinion, and with all the respect to the reviewer's comments, it is difficult to compare cisplatin doses used *in vitro* and *in vivo*. There are reports treating cancer cell lines *in vitro* with cisplatin at concentrations that range from 16 mM to 300 mM depending on the cell line. Based on the literature, concentrations higher than 100 mM are often required to observe effects in cancer cell lines in less than 24 h (Glorieux et al, 2011; Huang et al, 2003; Jamal et al, 2012; Park et al, 2012; Ru et al, 2011). As mentioned above, the purpose of using 100 mM cisplatin *in vitro* was to be able to find cell-death triggering mechanisms that are independent of the sensitivity of the cancer cell lines to cisplatin. Moreover, the observations obtained by using 100 mM cisplatin *in vitro* were validated *in vivo* by treating mice with a standard dose of cisplatin.

- I miss more direct evidence that p38alpha; is induced after cisplatin treatment. HSP27 phosphorylation is more indirect. Is p38alpha; still induced when 10microM of cisplatin are used?

The activation of p38 MAPK in response to cisplatin treatment has been reported by several groups, as reviewed in (Brozovic & Osmak, 2007) and (Sancho-Martinez et al, 2012). We provide direct evidence for the activation of p38 MAPK after cisplatin treatment in Fig 4C, 7C and Supporting Information Fig S1C.
Moreover, we have confirmed that 10 µM cisplatin can also induce p38 MAPK activation (see below).

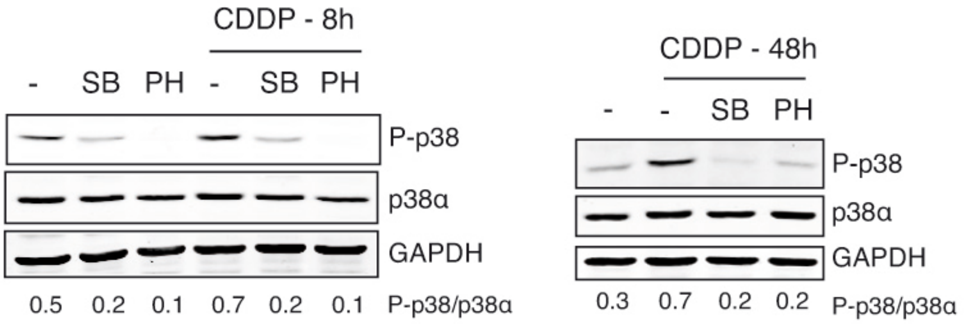

SW620 cells were pre-incubated for 2 h with SB203580 (SB, 10 μM) or PH-797804 (PH, 2 μM) and then treated with cisplatin (CDDP, 10 μM) for either 8 h or 48 h. Total cell lysates were analyzed with the indicated antibodies.

- For the RNAi experiments (Fig. 2 and Fig. 3) I miss deconvolution. Can the effect be reproduced with at least 2 independent shRNAs/siRNAs?

We are sorry for not clarifying in Materials and Methods that we did not use pools but individual siRNAs. Nonetheless, the implication of p38α as determined with two different chemical inhibitors was also validated using siRNAs and shRNAs (Fig 2A, 2B and New Fig 2C and 2D), and the results observed with shRNAs for JNK1/2 (Fig 2E) were confirmed with siRNAs (New Supporting Information Fig S2B).

- What is the effect on clonogenic assays when independent siRNAs against p38alpha are used?

We have now repeated the clonogenic assays using two different siRNAs for p38α and confirmed the previous results obtained with the p38 MAPK inhibitor (New Supporting Information Fig S4B). These results support that p38α is the major p38 MAPK family member contributing to the described mechanism.

- In vivo a different p38alpha/beta inhibitor is used than in vitro. Why?

The p38a and p38b inhibitor SB203580 has been routinely used in our laboratory and many others to inhibit p38 MAPK signaling *in vitro*. Although SB203580 can efficiently inhibit p38a and p38b activity, it has been reported to have several off-target effects (Bain et al, 2007). These effects can be controlled *in vitro* using RNAi but in mouse experiments may be problematic. For this reason, we decided to use for the *in vivo* experiments PH-797804, a potent inhibitor of p38a and p38b that is currently used in clinical trials (Goldstein et al, 2010; Hope et al, 2009).
As we agree that this point is important, we have now used PH-797804 to inhibit p38 MAPK in cancer cell lines. We show that PH-797804 recapitulates the effects observed with SB203580: increased apoptosis and phosphorylation of JNK when combined with cisplatin (New Supporting Information Fig S1B and S1C), which can be rescued with antioxidants (New Supporting Information Fig S3C).

- In vitro the authors genetically inhibit p38alpha as additional control to chemical inhibition. I miss an experiment in which p38alpha is also genetically inactivated in vivo.

We are studying the effect of genetically down-regulating p38a *in vivo*. Preliminary experiments confirm the results obtained using the chemical inhibitor and support an important role for p38a in breast cancer cell survival. However, this story needs to be further developed and should be the subject of a future publication.

- Is p38alpha activated in the in vivo model after cisplatin treatment?

This is an important point. The revised manuscript shows that the levels of total p38a protein do not change in tumors subjected to the different treatments but the levels of p38 MAPK phosphorylated in the activation sites are increased in tumors treated with cisplatin. These results indicate that p38 MAPK is activated *in vivo* after cisplatin treatment and successfully inhibited by PH797804 treatment (New Fig 7C).

- Do the authors manage to inhibit p38alpha successfully in vivo using PH-797804?

We have performed two experiments to show that p38 MAPK was inhibited *in vivo*. First, to confirm p38 MAPK inhibition we analyzed the level of phosphorylated HSP27, a downstream target of the p38 MAPK pathway, in tumor samples (New Fig 7C). In agreement with the phospho-p38 MAPK western blot, phosphorylated HSP27 was detected in tumors treated with cisplatin but not in those treated with cisplatin+p38 inhibitor. Second, since basal levels of phosphorylated HSP27 are not easily detectable, we subjected resected tumors to osmotic shock (300 mM NaCl, 15 min) right before the western blot analysis. This treatment induced effective p38 MAPK pathway activation in all cases except in tumors that have been treated with the p38 MAPK inhibitor (Supporting Information Fig S9A).

- Fig. 6A: The additional effect of p38alpha inhibition over cisplatin may be very small. Was the response monitored over a longer time than just 18 days? When do tumors relapse back to 200mm3? Why was the vehicle not added to cisplatin?

We agree with the Reviewer on the importance to monitor tumor behavior over a longer period of time as well as the relapse kinetics. In the revised manuscript, we have repeated the experiment using exactly the same treatments but monitoring the mice for up to 40 days (New Fig 7A). In addition to confirming the previous results, we observed that while tumors of mice treated only with cisplatin reached again 200 mm^3 in 20 days, those of mice treated with the combined therapy needed 40 days to reach 200 mm^3. These results indicate that tumors treated with the combined therapy re-grow significantly slower than the cisplatin-treated tumors (New Fig. 7A). Analysis of the relapsing 200 mm^3 tumors by Ki67 staining, confirmed the differences in proliferation status (New Fig 7D). To determine possible additional effects of p38 MAPK inhibition on the cisplatin response, we treated relapsing tumors coming from either cisplatin+vehicle or cisplatin+p38 inhibitor treatments with another single dose of cisplatin. Tumors previously treated with cisplatin+vehicle responded in exactly the same way as the first time that they were treated (New Supporting Information Fig S11). This response has been previously described by Borst and colleagues (Rottenberg et al, 2007; Rottenberg et al, 2012). Interestingly, tumors that were first treated with the combined therapy responded to the new dose of cisplatin and relapsed back to 200 mm^3 with very similar kinetics as the tumors treated with cisplatin+vehicle (New Supporting Information Fig S11). These observations support that the inhibition of p38 MAPK contributes to the delayed tumor re-growth observed in the combined therapy.
We have now clarified in the text that vehicle solution was also administrated by oral gavage every 24 h to the cisplatin treated animals. We always followed exactly the same procedure for vehicle as for the p38 MAPK inhibitor, to ensure that stress coming from animal manipulation did not interfere with the results.

- I miss quantification of the data presented in Fig. 6C and D. Moreover, The TUNEL pictures of 6C are hardly visible in the pdf copy (maybe due to low resolution pdf?). I think they require a higher quality picture.

We now quantified the data presented in the indicated figures. However, to accommodate the new experiment requested by the reviewer, the original Fig 6D and its quantification have been now moved to Supporting Information Fig S9C.
We have also replaced the TUNEL pictures of Fig 6C for new high-resolution images where the staining can be better appreciated (New Fig. 7B).

- Figure 6F: This is not useful, since this is apparently done on day 18, when tumors have shrunk after combination therapy. Small residual tumor nests may be miss-interpreted as adenoma. What about the tumor when it relapsed back to 200mm3?

We agree with the Reviewer that it is more relevant to analyze the tumors when they relapse back to 200 mm^3, and we have now included this analysis in the revised manuscript. We found that 200 mm^3 initial tumors showed heterogeneity with presence of hyperplasias, adenomas and carcinomas, being adenomas the most predominant stage. However, relapsing tumors showed a different distribution of the various tumoral stages depending on the treatment, even though they were all analyzed at the same size of 200 mm^3 (New Fig 7E). Of note, this analysis confirmed that tumors

treated with the combined therapy when relapsed back to the original 200 mm^3 (day 40) showed a higher degree of hyperplastic tissue and reduced Ki67 staining than tumors subjected to the other treatments (New Fig 7D).

- On page 9 the authors even state "a better prognosis could be expected in the event of a possible relapse after therapy termination." I do not find this serious. Usually relapsing tumors are more resistant. If the authors consider that there may be a better prognosis of relapsing tumors, why did they not test it?

We acknowledge that the indicated statement was not appropriate. However, as indicated above, we have now confirmed that relapsing tumors from the combined therapy re-grow significantly slower (New Fig 7A) show a reduction in the proliferation status measured by Ki67 staining (New Fig 7D) and contain a higher proportion of less-advanced tumoral stages, i.e. hyperplasias (New Fig 7E).
We have also treated relapsing tumors coming from cisplatin+vehicle or cisplatin+ p38 inhibitor treatments with another single dose of cisplatin (New Supporting Information Fig S11). Both groups responded again to cisplatin treatment with more or less the same kinetics, indicating that the tumors did not became resistant to the therapy and also that p38 MAPK inhibition was contributing to the delayed tumoral growth observed in the combined treatment with cisplatin.
We have edited the text to discuss the new information and have also removed the indicated sentence, as we have no evidence that a better prognosis could be expected in the relapsing tumors.

- The authors mention in the introduction that it is already known that cisplatin resistance is linked to increased MAPK signaling. Overall it is somewhat unclear to me what is really new to justify publication in EMM.

This point was addressed above. The sentence in the Introduction was misleading and we apologize for not being clearer. In the first paper that we cited (Galan-Moya et al, 2011), cell lines derived from non-small cell lung cancer or head and neck carcinomas are used to show that sensitivity to cisplatin is associated with activation of p38 MAPK. Intriguingly, this paper also shows that cancer cell lines with high basal levels of p38 MAPK activity, which correlate with high expression of the MKK3 upstream activator, tend to be more resistant to cisplatin. The authors propose that resistance correlates with the inability to further activate p38 MAPK in response to cisplatin. This manuscript provides no information on the mechanism by which p38 MAPK signaling could impinge on cisplatin sensitivity or resistance. We have now clarified this in the revised manuscript. In the second paper (Chung et al, 2012), knockdown of galectin-1 in lung cancer cells lines is reported to enhance sensitivity to cisplatin, which correlates with downregulation of ERK, p38 MAPK and NFkB as well as attenuated expression of COX-2. After careful re-evaluation of this paper, we have not found any experiment to confirm the implication of p38 MAPK in the reported effects and we have therefore removed the reference from the revised manuscript.
Our work provides evidence for a new mechanism that explains why inhibition of p38 MAPK sensitizes cancer cells to apoptosis. We show that p38 MAPK maintains low basal levels of ROS and phosphorylated JNK in cancer cells. As a consequence, inhibition of p38 MAPK enhances the pool of phosphorylated JNK, which in turn induces apoptosis of the cancer cell more efficiently after cisplatin treatment. This applies to cancer cells that have different levels of basal p38 MAPK activity. Moreover, we provide to our knowledge the first *in vivo* evidence that inhibition of p38 MAPK sensitizes tumor cells to cisplatin-induced apoptosis.

Referee #3 (Remarks):

1. Figure 1B. This figure needs the data for 'SB alone' to be included.

We have now changed the figure in the revised manuscript to include the data for SB203580 alone.

2. Figure 1D needs the 'control' and 'SB alone' data included so that the reader can clearly see the degree of synergy or additivity between the treatment regimens.

In the revised manuscript, Fig 1D has been removed and is replaced by more complete clonogenic experiments including inhibitors (New Fig 3E) and siRNAs (New Supporting Information Fig S4B). The requested controls are shown in New Supporting Information Fig S4A.

3. Why does the JNK inhibitor SP600125 inhibit JNK phosphorylation in Figure 1F? This drug is an inhibitor of JNK not MKK4 or MKK7

We agree with the reviewer that as an inhibitor of JNK activity, SP600125 should in principle not affect JNK phosphorylation. However, MAPK pathways are often involved in complex feedback loops. Thus, the observed inhibition of JNK phosphorylation might happen because we always do our experiments in the presence of serum, a condition where JNK can induce cytokine production, like TNFα, which in turn would activate JNK. Therefore, inhibition of JNK will not only block downstream phosphorylation events but, as a consequence of feedback loops, could also reduce JNK phosphorylation. The dephosphorylation of JNK after SP600125 treatment has been previously reported by others (Gururajan et al, 2005; Park et al, 2012; Vasilevskaya et al, 2004). We have also observed similar effects using inhibitors of p38 MAPK, which sometimes induce p38 MAPK de-phosphorylation depending on the cell line used and the treatment, consistent with the idea of indirect effects.

4. The ability of JNK inhibition (SP600125 or shRNA) to overcome the cell death observed with CDDP+SB is only shown using PARP cleavage (Figure 1F and 2D), a qualitative assay. This is a very important part of the study and needs to be demonstrated with robust quantitative assays (DNA oligonucleosomes, sub-G1 or Annexin-V) and appropriate statistics.

We thank the Reviewer for this suggestion. In the revised the manuscript, we have used quantitative assays to show that siRNAs against JNK1 and JNK2 can reduce the levels of cell death in cancer cells treated with cisplatin and the p38 MAPK inhibitor (New Supporting Information Fig S2B).

5. The ability of ROS scavengers to protect cells from CDDP+SB is also only shown by PARP cleavage (Figure 3D and 3F) and needs to be confirmed with quantitative assays.

We have now performed quantitative assays to confirm that antioxidants can protect from the cell death induced by the treatment with cisplatin and the p38 MAPK inhibitor (New Fig 3D).

6. It is very interesting that CDDP and SB cooperate to activate JNK and promote cell death in SW620 cells as these cells have a mutation in MKK4 that generates a truncated non-functional protein. This suggests that JNK activation is exclusively via MKK7 in these cells? A MKK7 siRNA experiment here would add significant mechanistic insight.

This is an interesting suggestion and we have tried to do the experiment several times but have failed to detect significant siRNA-mediated down-regulation of MKK7 in SW620 cells. Nevertheless, one would expect that the downregulation of MKK7 in cells that do not express MKK4 should impair JNK phosphorylation, unless this is the result of autophosphorylation or some other non-canonical mechanism of activation.

7. Figure 5C. The blots indicate that DUSP16 knockdown also increases JNK phosphorylation - especially the p54 forms. Might this explain why DUSP8 is not the key regulator in other cells (Fig S6)?

We have repeated this experiment several times and consistently find that DUSP8 knockdown increases JNK phosphorylation in MCF7 cells more potently than the DUSP16 knockdown. However, neither DUSP8 nor DUSP16 seem to play a major role in the regulation of basal JNK phosphorylation levels in other cancer cell lines (Supporting Information Fig S7). We therefore conclude that basal JNK activity levels are probably controlled by different ROS-sensitive phosphatases depending on the tumor cell type. We have re-written the text in Results and Discussion to clarify this point.

Minor points
1. Penultimate line of page 7. Oxidation spelt incorrectly ('inactivation by oxidation').

This has been corrected in the revised manuscript.

References

Bain J, Plater L, Elliott M, Shpiro N, Hastie CJ, McLauchlan H, Klevernic I, Arthur JS, Alessi DR, Cohen P (2007) The selectivity of protein kinase inhibitors: a further update. Biochem J 408: 297-315
Brozovic A, Osmak M (2007) Activation of mitogen-activated protein kinases by cisplatin and their role in cisplatin-resistance. Cancer Lett 251: 1-16
Chung LY, Tang SJ, Sun GH, Chou TY, Yeh TS, Yu SL, Sun KH (2012) Galectin-1 promotes lung cancer progression and chemoresistance by upregulating p38 MAPK, ERK, and cyclooxygenase-2. Clin Cancer Res18: 4037-4047
Galan-Moya EM, de la Cruz-Morcillo MA, Llanos Valero M, Callejas-Valera JL, Melgar-Rojas P, Hernadez Losa J, Salcedo M, Fernandez-Aramburo A, Ramon y Cajal S, Sanchez-Prieto R (2011) Balance between MKK6 and MKK3 mediates p38 MAPK associated resistance to cisplatin in NSCLC. PloS One 6: e28406
Glorieux C, Dejeans N, Sid B, Beck R, Calderon PB, Verrax J (2011) Catalase overexpression in mammary cancer cells leads to a less aggressive phenotype and an altered response to chemotherapy. Biochem Pharmacol 82: 1384-1390
Goldstein DM, Kuglstatter A, Lou Y, Soth MJ (2010) Selective p38alpha inhibitors clinically evaluated for the treatment of chronic inflammatory disorders. J Med Chem 53: 2345-2353
Gururajan M, Chui R, Karuppannan AK, Ke J, Jennings CD, Bondada S (2005) c-Jun N-terminal kinase (JNK) is required for survival and proliferation of B-lymphoma cells. Blood 106: 1382-1391
Hope HR, Anderson GD, Burnette BL, Compton RP, Devraj RV, Hirsch JL, Keith RH, Li X, Mbalaviele G, Messing DM, Saabye MJ, Schindler JF, Selness SR, Stillwell LI, Webb EG, Zhang J, Monahan JB (2009) Anti-inflammatory properties of a novel N-phenyl pyridinone inhibitor of p38 mitogen-activated protein kinase: preclinical-to-clinical translation. J Pharmacol Exp Ther 331: 882-895
Huang HL, Fang LW, Lu SP, Chou CK, Luh TY, Lai MZ (2003) DNA-damaging reagents induce apoptosis through reactive oxygen species-dependent Fas aggregation. Oncogene 22: 8168-8177
Jamal MH, Ch'ng WC, Yusoff K, Shafee N (2012) Reduced Newcastle disease virus-induced oncolysis in a subpopulation of cisplatin-resistant MCF7 cells is associated with survivin stabilization. Cancer Cell Int 12: 35
Park IJ, Kim MJ, Park OJ, Choe W, Kang I, Kim SS, Ha J (2012) Cryptotanshinone induces ER stress-mediated apoptosis in HepG2 and MCF7 cells. Apoptosis 17: 248-257
Rottenberg S, Nygren AO, Pajic M, van Leeuwen FW, van der Heijden I, van de Wetering K, Liu X, de Visser KE, Gilhuijs KG, van Tellingen O, Schouten JP, Jonkers J, Borst P (2007) Selective induction of chemotherapy resistance of mammary tumors in a conditional mouse model for hereditary breast cancer. Proc Natl Acad Sci USA 104: 12117-12122
Rottenberg S, Vollebergh MA, de Hoon B, de Ronde J, Schouten PC, Kersbergen A, Zander SA, Pajic M, Jaspers JE, Jonkers M, Loden M, Sol W, van der Burg E, Wesseling J, Gillet JP, Gottesman MM, Gribnau J, Wessels L, Linn SC, Jonkers J, Borst P (2012) Impact of intertumoral heterogeneity on predicting chemotherapy response of BRCA1-deficient mammary tumors. Cancer Res 72: 2350-2361
Ru P, Steele R, Hsueh EC, Ray RB (2011) Anti-miR-203 Upregulates SOCS3 Expression in Breast Cancer Cells and Enhances Cisplatin Chemosensitivity. Genes Cancer 2: 720-727
Sancho-Martinez SM, Prieto-Garcia L, Prieto M, Lopez-Novoa JM, Lopez-Hernandez FJ (2012) Subcellular targets of cisplatin cytotoxicity: an integrated view. Pharmacol Ther 136: 35-55
Vasilevskaya IA, Rakitina TV, O'Dwyer PJ (2004) Quantitative effects on c-Jun N-terminal protein kinase signaling determine synergistic interaction of cisplatin and 17-allylamino-17-demethoxygeldanamycin in colon cancer cell lines. Mol Pharmacol 65: 235-243

3rd Editorial Decision 19 August 2013

Thank you for the submission of your revised manuscript to EMBO Molecular Medicine. We have now received the enclosed reports from the referees that were asked to re-assess it. As you will see the reviewers are now globally supportive and I am pleased to inform you that we will be able to accept your manuscript pending the following final amendments:

-Referee 2 suggests to include the Reviewer figure in the manuscript. I would agree with this referee that the data contained in this figure is important as readers may also wonder about the drug concentrations used. However, I would leave it to you whether to add it to the Supplementary Information as suggested or within the point-by-point response, assuming you will agree to publish this figure online as part of our Review Process File (please see below).

-We now encourage the publication of source data, particularly for electrophoretic gels and blots, with the aim of making primary data more accessible and transparent to the reader. Would you be willing to provide a single PDF file comprising the original, uncropped and unprocessed scans of all or key gels used in the figures? These should be labeled with the appropriate figure/panel number, and should have molecular weight markers; further annotation could be useful but is not essential. This PDF will be published online with the article as a supplementary "Source Data" file. If you have any questions regarding this just contact me.

-Figures: Fig 1B, 1E have misaligned boxes. Fig6A has too much contrast

Please submit your revised manuscript within two weeks. I look forward to seeing a revised form of your manuscript.

***** Reviewer's comments *****

Referee #1 (Comments on Novelty/Model System):

The manuscript by Pereira et al has now been improved substantially.

Referee #1 (Remarks):

In my view the authors have appropriately addressed all the reviewer's comments.

Referee #2 (Remarks):

I am fine with the responses to my comments. I agree that comparisons between in vitro and in vivo are difficult. Nevertheless, to me these high cisplatin concentrations that are used in vitro remain unrealistic, and therefore it is questionable whether they help to find something useful, or whether they provide artifacts which are not worth pursuing. I leave this to the editor. Independent of the editor's decision, I think that the data using a lower cisplatin concentration (reviewer figure) should be included as Suppl. data.

Minor point:

Response to my point 2: I do not find it appropriate to remove these publications. They should be discussed with the present data.

2nd Revision - authors' response 26 August 2013

We have addressed the final amendments as follows:

- The point-by-point responses to be published online now include the two figures with data on the effects of 10 μM cisplatin (pages 3 and 4). We have also added a sentence in page 2 (line 13) and page 8 (line 16) to clarify that we do discuss in Introduction one of the original publications mentioned in point 2, while the other one was removed due to lack of evidence for the implication of p38 MAPK in the reported effects. Moreover, we have added a sentence in page 4 (line 8) to emphasize that, in spite of the reviewer’s skepticism, the in vitro data obtained by using 100 μM cisplatin were validated in vivo by treating mice with a standard dose of cisplatin.

- We have included a PDF file (labeled Source Data) with the original scans for the majority of the gels/blots used in the figures. Molecular weight markers have been included in all cases. The blots were often cut before hybridization and different parts were probed with various antibodies, as indicated.

- Figures: we have included new versions of Fig 1 (with aligned boxes in 1B and 1E), and of Fig 5 (indicating that a lane was removed from the blot of 5B, as shown in the original scan, because it was not relevant for the point illustrated).

Regarding Fig 6A, the experiments were performed several years ago and we have not been able to locate the original file for the image provided. However, we found raw data for other experiments showing the same result (inhibition of JNK phosphatases in lysates of cells treated with SB203580) although sampling was taken at slightly different times. Therefore, we propose to replace the original panel 6A, so that we can include as Source Data the uncropped scans for both the Coomassie stained gel and its autoradiography.

- Manuscript text: we have added a sentence to the legend to Fig 5B indicating that a lane was removed from the blot. We have also corrected a few grammatical and technical mistakes, which we overlooked in the previous revision.

We hope that you will find these modifications satisfactory and will be able to accept our manuscript for publication in EMM.
