# Supplementary material for: Inhibition of p38 MAPK sensitizes tumour cells to cisplatin-induced apoptosis mediated by reactive oxygen species and JNK
Source: EMBO Mol Med. 2013 Sep 24;5(11):1759–74. doi: 10.1002/emmm.201302732 (PMC3840490; doi:10.1002/emmm.201302732)
Supplement: Supplementary file 2 [file emmm0005-1759-SD2.pdf]

SUPPORTING INFORMATION

Inhibition of p38 MAPK sensitizes tumor cells to cisplatin-induced apoptosis mediated by reactive oxygen species and JNK

Lorena Pereira, Ana Igea, Begoña Canovas, Ignacio Dolado and Angel R. Nebreda

Table of Content

Table S1

Table S2

Figure S1

Figure S2

Figure S3

Figure S4

Figure S5

Figure S6

Figure S7

Figure S8

Figure S9

Figure S10

Figure S11

Figure S12

Table S1. Antioxidant enzyme-encoding genes regulated by p38 MAPK in cancer cells

RefSeq	Name	Array	MCF7	HT-29
NM_002083	GPX2- Glutathione peroxidase 2	-1.91	-1.38	1.83
NM_002084	GPX3-Glutathione peroxidase 3	-1.49	-1.07	-1.31
NM_001509	GPX5- Glutathione peroxidase 5	-1.96	-2.16	-1.50
NM_000637	GSR- Glutathione reductase	-1.70	-1.17	1.09
NM_006151	LPO- Lactoperoxidase	-1.88	1.50	- 5.56
NM_000963	PTGS2/COX2 - Prostaglandin-endoperoxide synthase 2	-5.87	-4.09	-1.53
NM_144651	PXDNL- Peroxidasin homolog	-1.86	-9.52	-1.45
NM_032243	TXNDC2- Thioredoxin domain containing 2	-3.15	-1.91	-2.35

The expression of eight candidate genes identified in the array was validated by RT-PCR in breast MCF7 and colon HT-29 cancer cells. Numbers indicate fold changes in the expression levels of the genes upon incubation of the cells with SB203580 to inhibit p38 MAPK signaling. Genes downregulated at least 1.5 fold in both cell lines are shadowed

Table S2. Sequences of primers used for RT-PCR analysis

Refseq	Gene	Primers sequences
NM_002083	GPX2	FW: 5'- CCTCCCCACCCCTCTAATAG - 3'
		RV: 5'- TCTACCTTCTCCCCATCCAG - 3'
NM_002084	GPX3	FW: 5'- TGTCAATGGAGAGAAAGAGCAG - 3'
		RV: 5'- TCTCAAAGTTCCAGCGGATG - 3'
NM_001509	GPX5	FW: 5'- ACATCCTGGCGTACTTGAAG - 3'
		RV: 5'- GGGAGAGCAAGTCATTAGGTG - 3'
NM_000637	GSR	FW: 5'- CAGGGACTTGGGTGTGATG - 3'
		RV: 5'- ACGTGTCTCCTGGTTCTCA - 3'
NM_006151	LPO	FW: 5'- TGAAAAGACAAGGCACTGGG - 3'
		RV: 5'- TGAAGCAAGATGAGGGAAGC - 3'
NM_000963	PTGS2	FW: 5'- ACAGGCTTCCATTGACCAG - 3'
		RV: 5'- TCACCATAGAGTGCTTCCAAC - 3'
NM_144651	PXNDL	FW: 5'- CCAAGTGATTCCCCAGAGAAG -3 '
		RV: 5'- GTTGCTTAAGTCAGTGGTTTCC - 3'
NM_032243	TXNDC2	FW: 5'- TGATGCCAGTGAATGCGTAG - 3'
		RV: 5'- CGTTGCTGGACAGGACTAG - 3'
NM_002827	PTPN1	FW: 5'- AGAAGGACGAGGACCATGCAC - 3'
		RV: 5'- AGTGGAGGAGGGTCAGGCTAT - 3'
NM_002835	PTPN12	FW: 5'- GATGGTGCTGTGACCAGGAAC - 3'
		RV: 5'- TCATGTCCATTCTGAAGGTGG - 3'
NM_004420	DUSP8	FW: 5'- GATGACGCAAAATGGAATAAGC - 3'
		RV: 5'- CTTCACGAACCTGTAGGCG - 3'
NM_144729	DUSP10	FW: 5'- TGAACATCGGCTACGTCATC - 3'
		RV: 5'- TGGTGTAAGGATTCTCGGTG - 3'
NM_030640	DUSP16	FW: 5'- CAGAATGGGATTGGTTATGTG - 3'
		RV: 5'- TAGGCGATAGCGATGGTGG - 3'

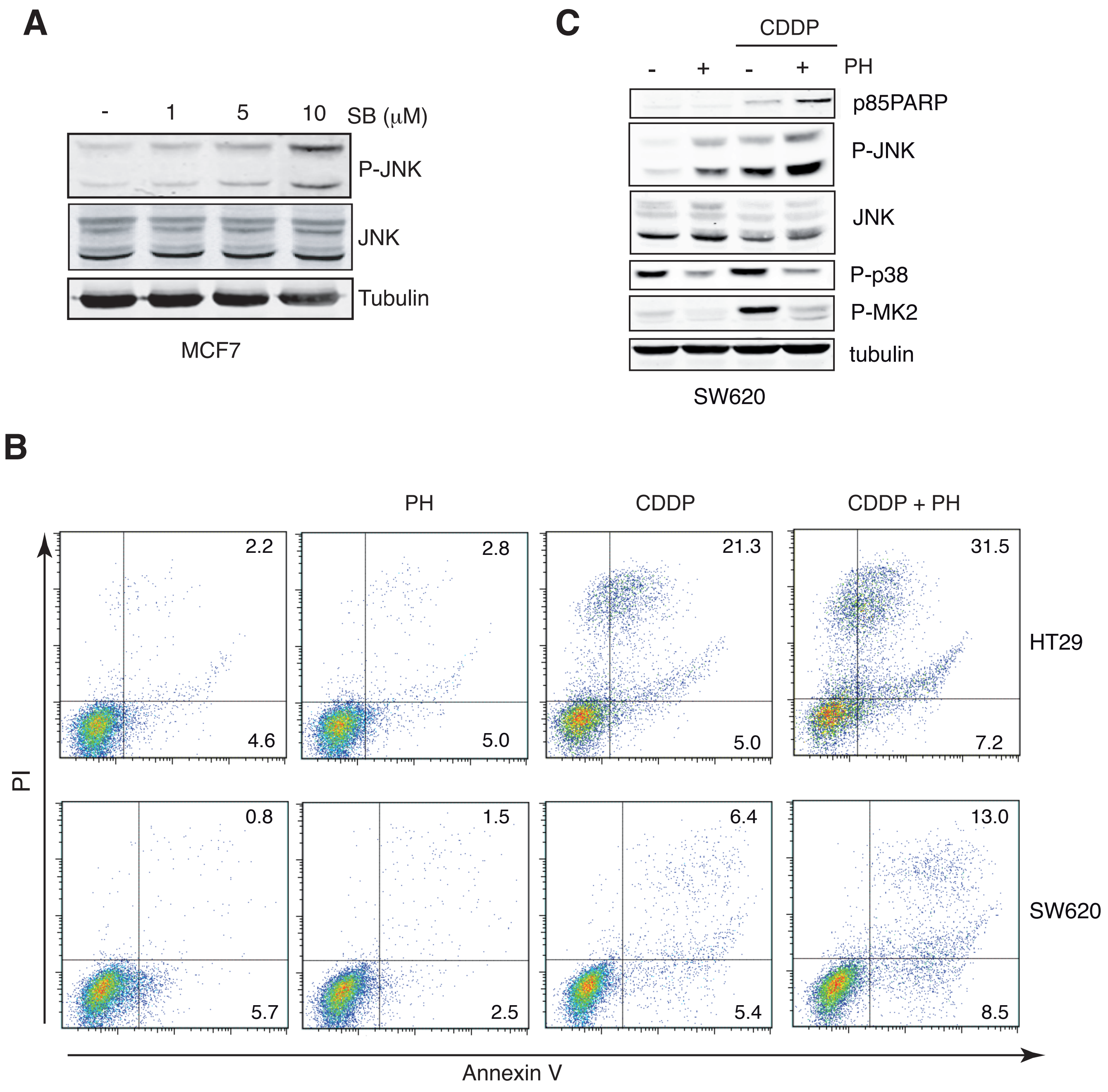

Figure S1.

A. MCF7 cells were treated with increasing concentrations of SB203580 (SB, 1- 10 µM) for 6 h. Total cell lysates were analyzed by immunoblotting with the indicated antibodies.

B. HT-29 and SW620 cells were pre-incubated for 2 h with PH-797804 (PH, 2 µM) and then treated with cisplatin (CDDP, 100 µM) for 24 h. Cell death was measured with propidium iodide (PI) and Annexin V staining. The percentages of apoptotic cells are indicated.

C. SW620 cells were incubated overnight with PH-797804 (PH, 2 µM) and then treated for 8 h with cisplatin (CDDP, 100 µM). Total cell lysates were analyzed by immunoblotting with the indicated antibodies.

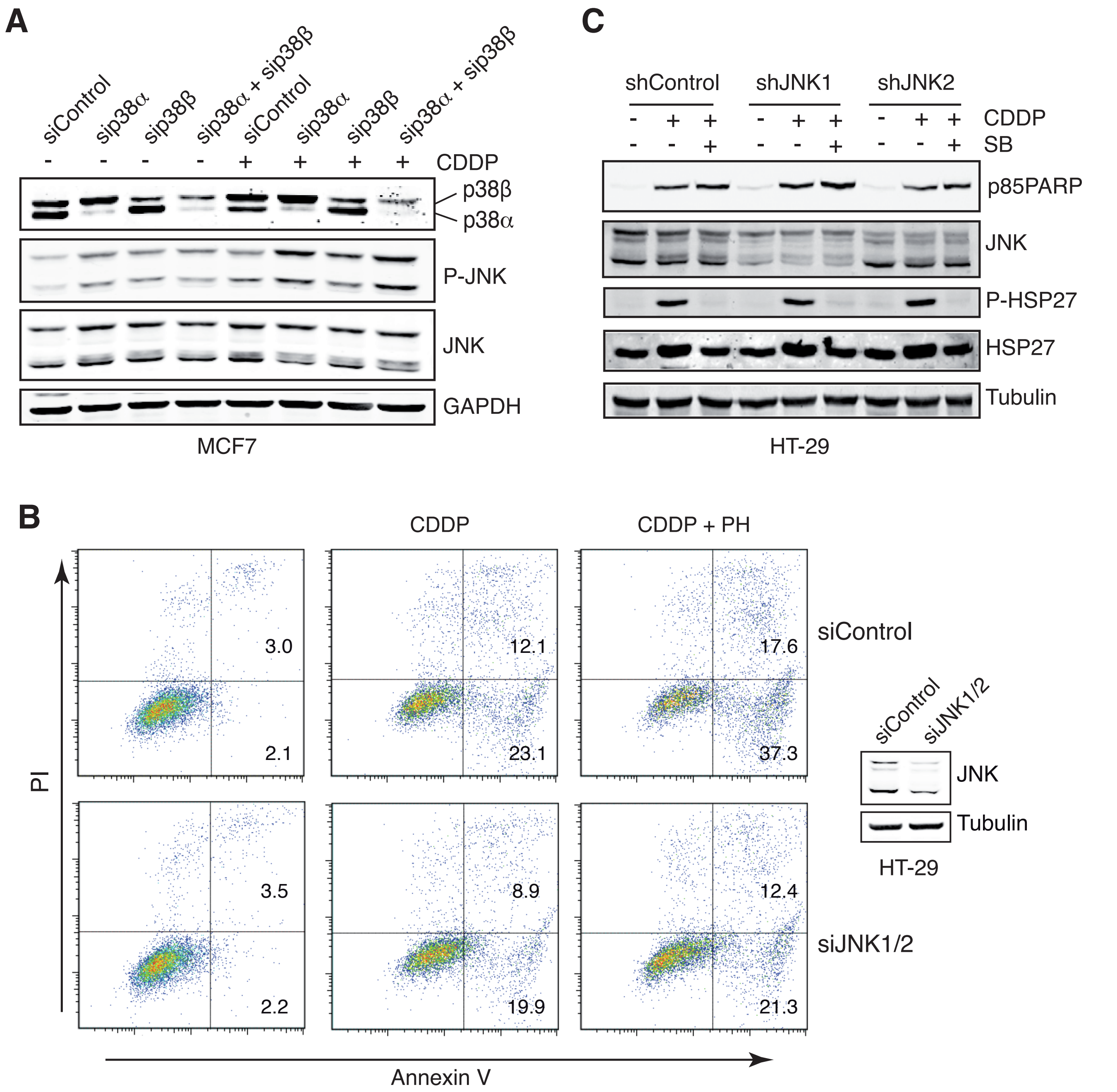

Figure S2.

A. MCF7 cells were transfected with siRNAs (50 nM) either control or against p38α, p38β or both together. After 48 h, cells were treated for 8 h with cisplatin (CDDP, 100 μM). Total lysates were analyzed by immunoblotting with the indicated antibodies. The upper blot was incubated first with the p38β antibody and then with the p38α antibody.

B. HT-29 cells were transfected with siRNAs (50 nM) either control or against JNK1/2. After 48 h, cells were incubated for 2 h in the presence or absence of PH-797804 (PH) and then treated for 24 h with cisplatin (CDDP, 100 μM). Cell death was quantified by staining with propidium iodide (PI) and Annexin V. Total cell lysates were analyzed by immunoblotting with the indicated antibodies (right panel).

C. HT-29 cells were infected with lentiviruses expressing shRNAs against JNK1 or JNK2 or a non-targeting control. Pools of cells were incubated overnight in the presence or absence of SB203580 (SB, 10 μM) and then treated for 8 h with cisplatin (CDDP, 100 μM). Total cell lysates were analyzed by immunoblotting with the indicated antibodies.

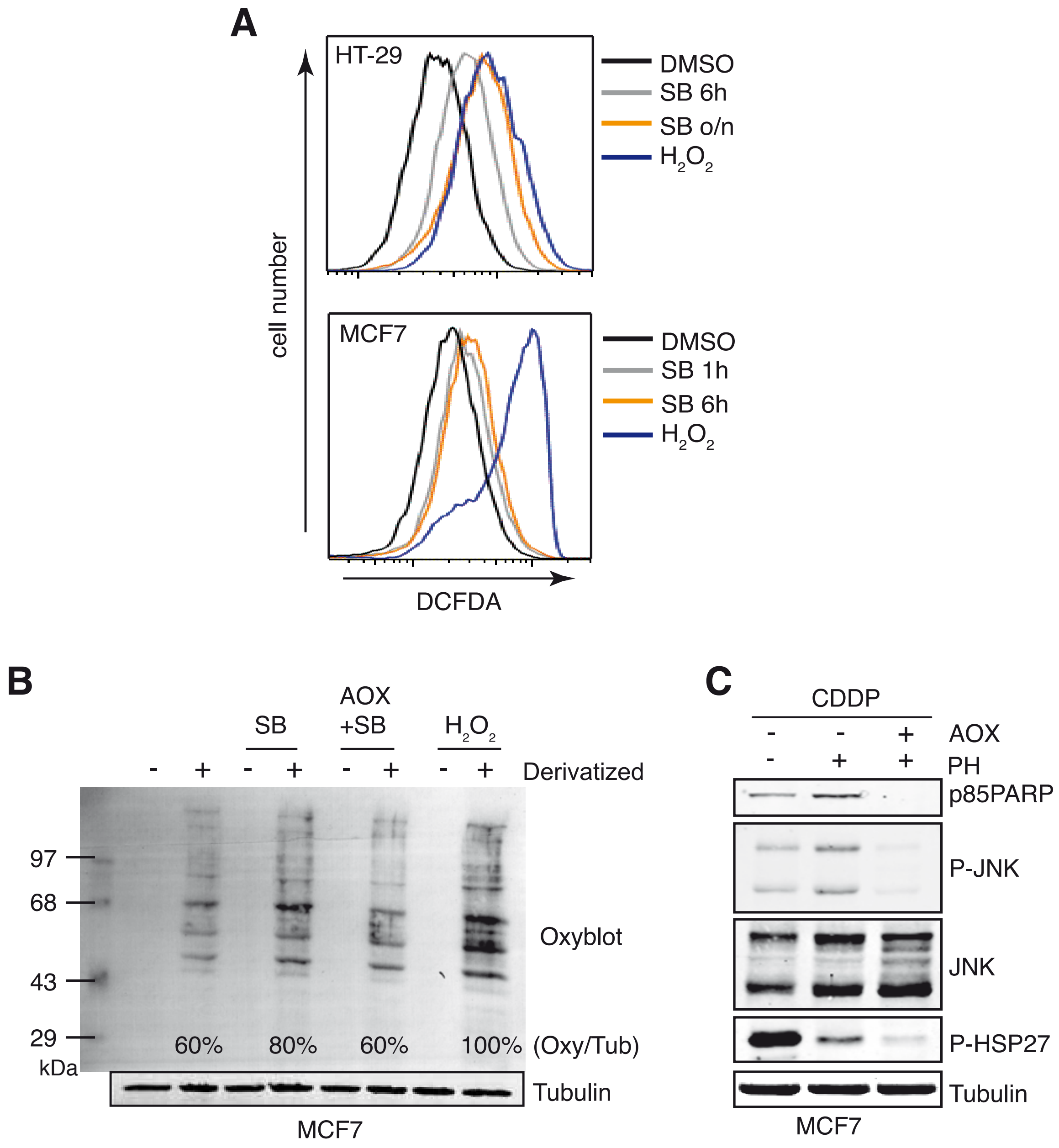

Figure S3.
A. HT-29 and MCF7 cells were incubated for the indicated times with SB203580 (SB, 10 μM), overnight with DMSO, or 10 min with H_2O_2 (5 mM). ROS levels were measured with DCFDA by flow cytometry.
B. MCF7 cells were pre-treated for 1 h with a mixture of NAC and GSH antioxidants (AOX) and then incubated overnight with SB203580 (SB, 10 μM), or were treated with 5 mM H_2O_2 for 1 h, as indicated. Cell lysates were analyzed by immunoblotting using Oxyblot and the total protein oxidation signal per lane was quantified using tubulin as a reference.
C. MCF7 cells were pre-treated for 1 h with antioxidants (AOX) as in (**B**), followed by overnight incubation with PH-797804 (PH, 2 μM) and then treated with cisplatin (CDDP, 100 μM) for 8 h. Total cell lysates were analyzed by immunoblotting with the indicated antibodies.

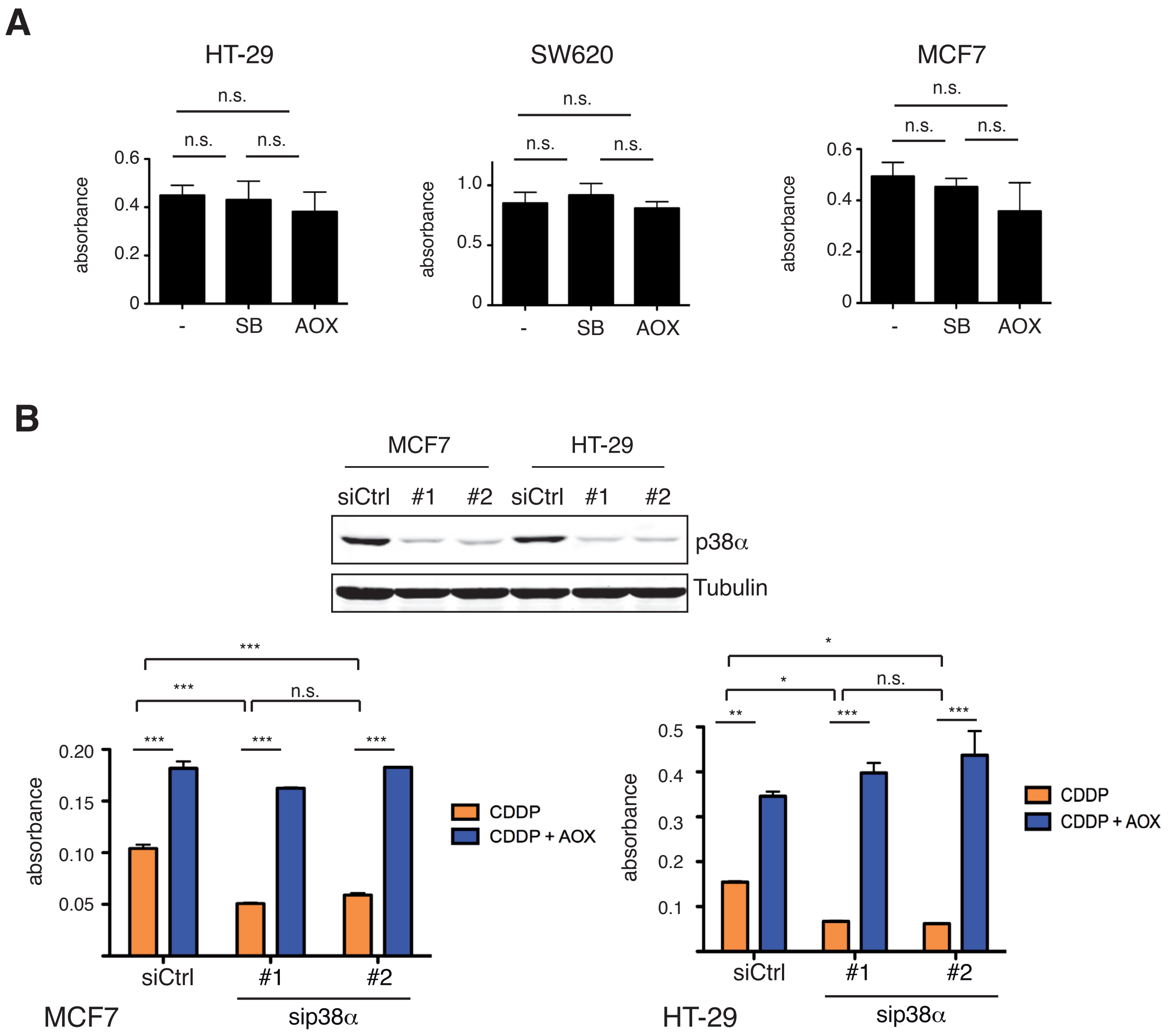

Figure S4.
A. Cells were treated for 1 h with a mixture of NAC and GSH antioxidants (AOX) or incubated overnight with SB203580 (SB, 10 µM) and then plated for clonogenic assays. Colonies were dissolved in methanol and the absorbance reads (540 nm) are represented in the bar diagrams.
B. Cells were incubated for 48 h either with two different siRNAs against p38α (#1 and #2) or with a scrambled siRNA (siCtrl) as a control. Total cell lysates were analyzed by immunoblotting with the indicated antibodies (upper blot). For clonogenic assays, siRNA-treated cells were treated for 1 h with AOX as in (**A**) followed by 1 h incubation with cisplatin (CDDP) and then plated. Colonies were dissolved in methanol and the absorbance reads (540 nm) are represented in the bar diagrams.

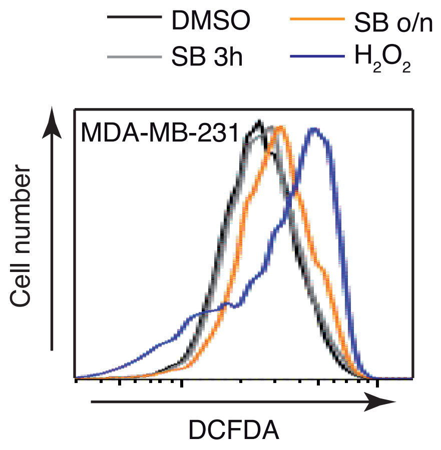

Figure S5. MDA-MB-231 cells were incubated for the indicated times with SB203580 (SB, 10 µM), overnight with DMSO or 10 min with H_2O_2 (5 mM). ROS levels were measured with DCFDA by flow cytometry

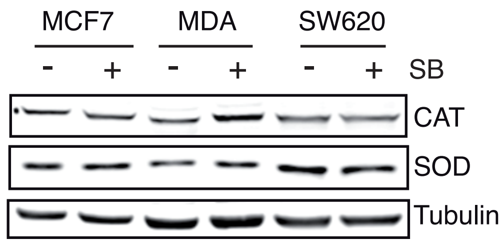

Figure S6.
MCF7, MDA-MB-231 and SW620 cancer cell lines were treated overnight with SB203580 (SB, 10 µM). Total cell lysates were analyzed by immunoblotting with the indicated antibodies. CAT : catalase; SOD: superoxide dismutase.

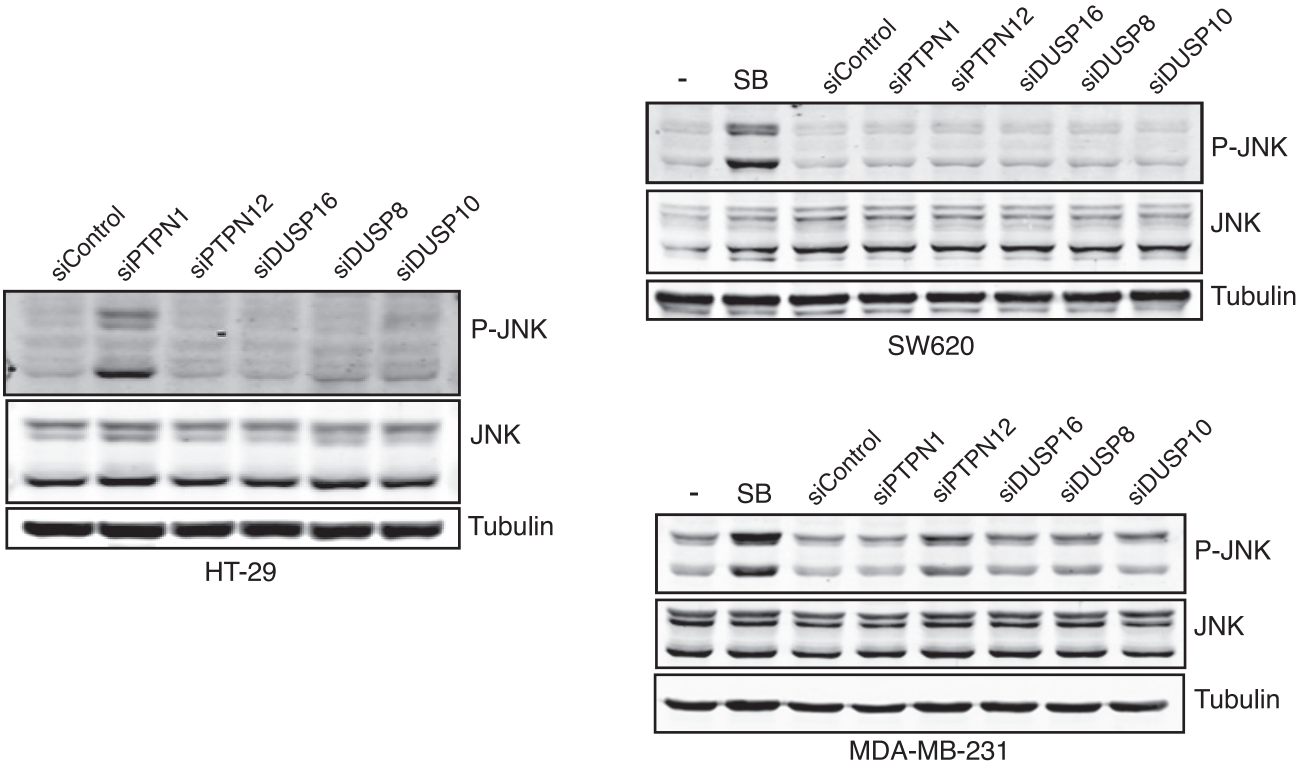

Figure S7.
The indicated cancer cell lines were transfected with siRNAs (50 nM) against PTPN1, PTPN12, DUSP16, DUSP8 and DUSP10, or with a scramble control, and 48 h later total cell lysates were analyzed by immunoblotting with the indicated antibodies. As a positive control, cells were incubated overnight with SB203580 (SB, 10 μM) before immunoblotting.

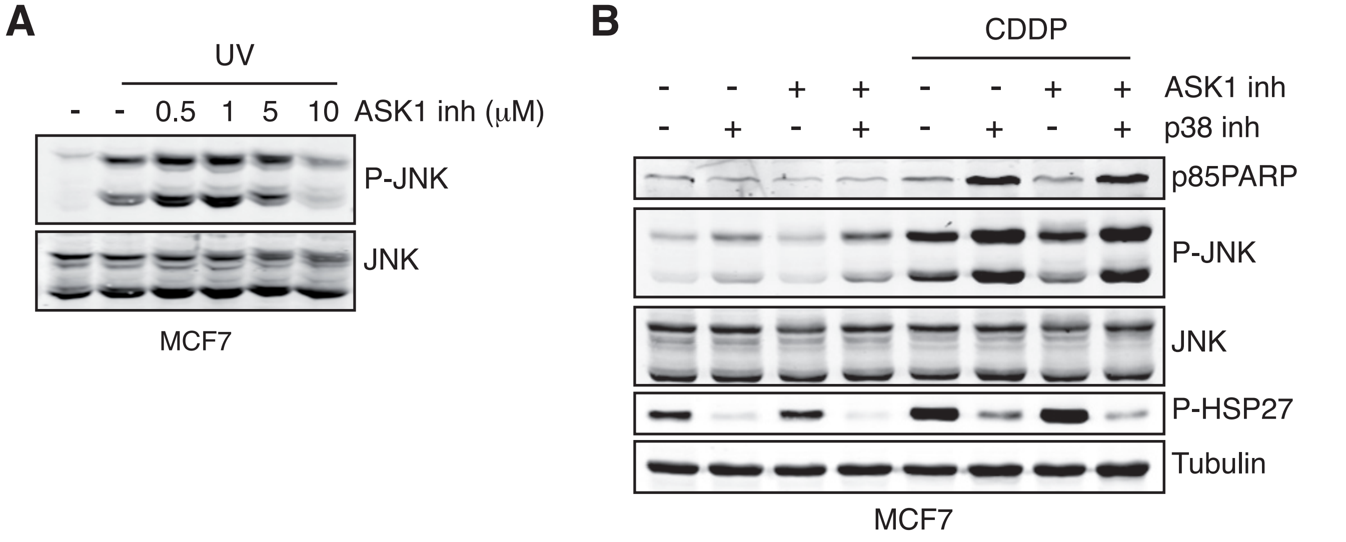

Figure S8.
A. MCF7 cells were incubated with the indicated concentrations of ASK1 inhibitor (ASK1 inh) overnight and then irradiated with UV (50 J/m^2) followed by 30 min incubation at 37ºC. Total cell lysates were analyzed by immunoblotting with the indicated antibodies.
B. MCF7 cells were incubated overnight with SB203580 (p38 inh, 10 μM) and/or ASK1 inhibitor (10 μM) followed by cisplatin (CDDP, 100 μM) for 8 h. Total cell lysates were analyzed by immunoblotting with the indicated antibodies.

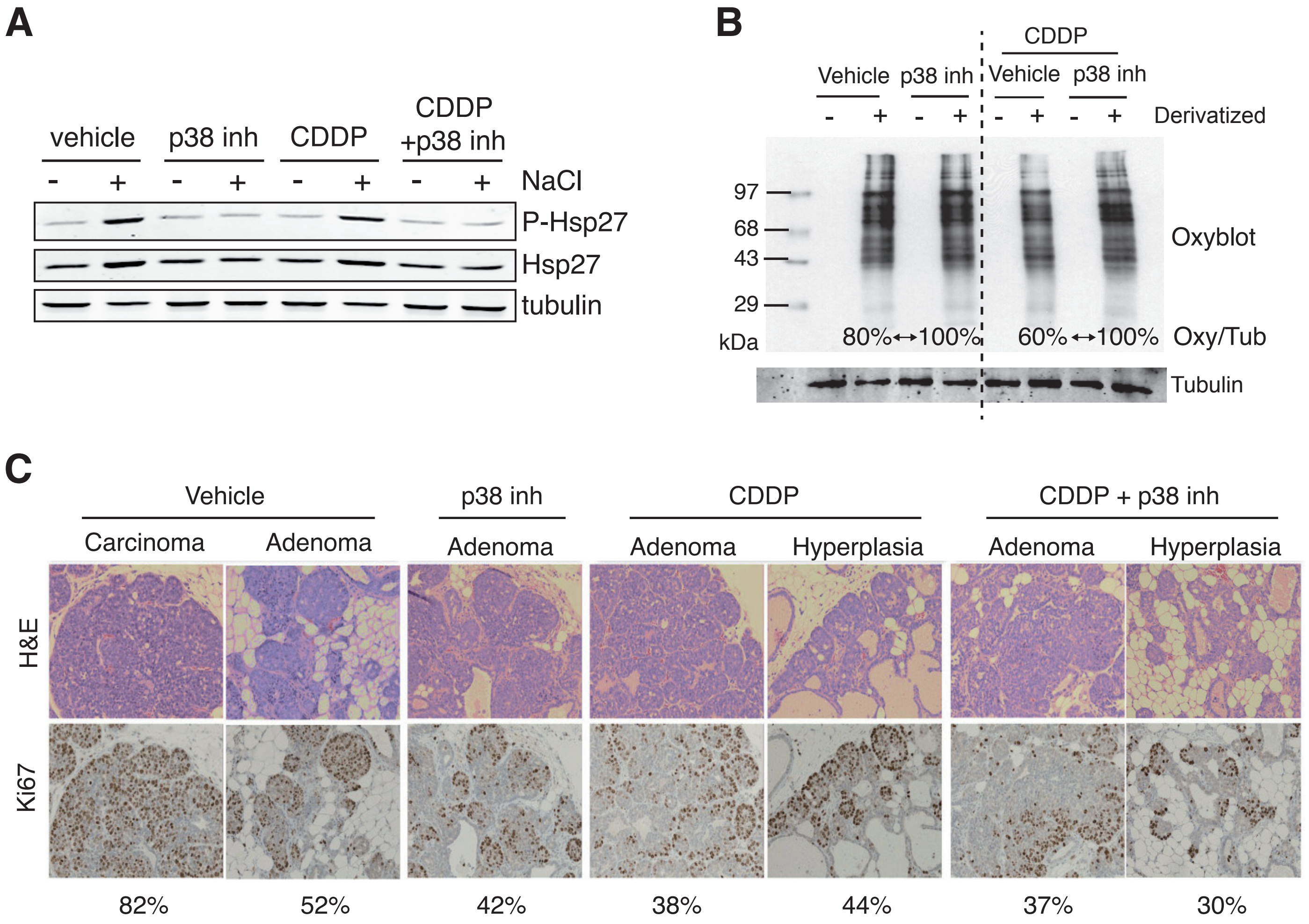

Figure S9.

A. Breast tumors obtained from different treatments were incubated, immediatly after resection, for 15 min in a solution of NaCl (300 mM) and then snap frozen. Total tissue lysates were analyzed by immunoblotting with the indicated antibodies.

B. Total lysates from breast tumors at day 7 were analyzed by immunoblotting using Oxyblot. The total protein oxidation signal per lane was quantified with tubulin as a reference. Results were confirmed using three mice per condition.

C. H&E and Ki67 staining of breast tumors analyzed at day 7. Quantifications of Ki67 staining are indicated. Images are 20x

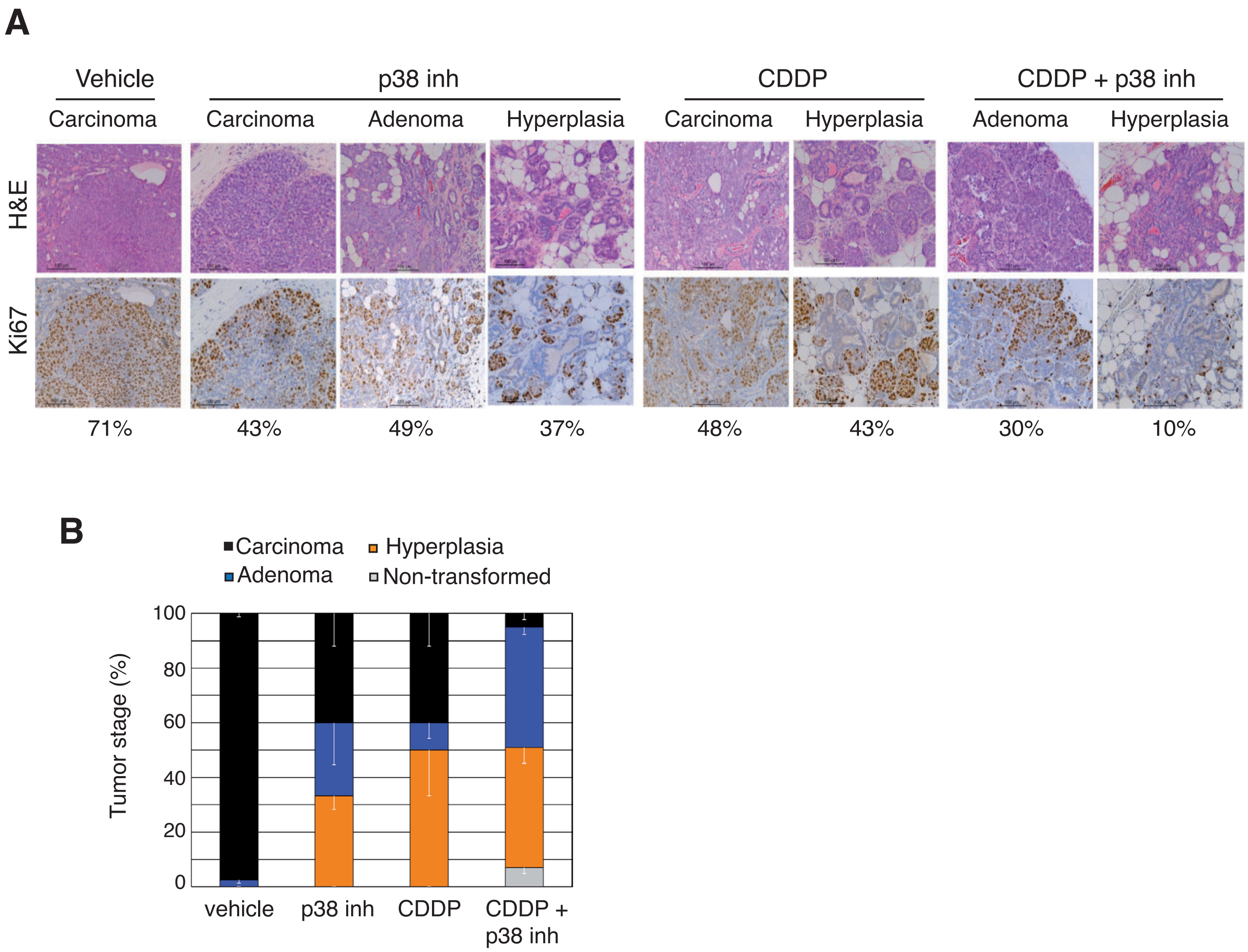

Figure S10.
A. H&E and Ki67 staining of breast tumors collected at day 18. Images shown are 20x. Quantifications of Ki67 staining are indicated.
B. H&E stained sections of breast tumors collected at day 18 were analyzed under the microscope in blinded fashion. Samples were classified as carcinoma, adenoma, hyperplasia and normal tissue (non-transformed).

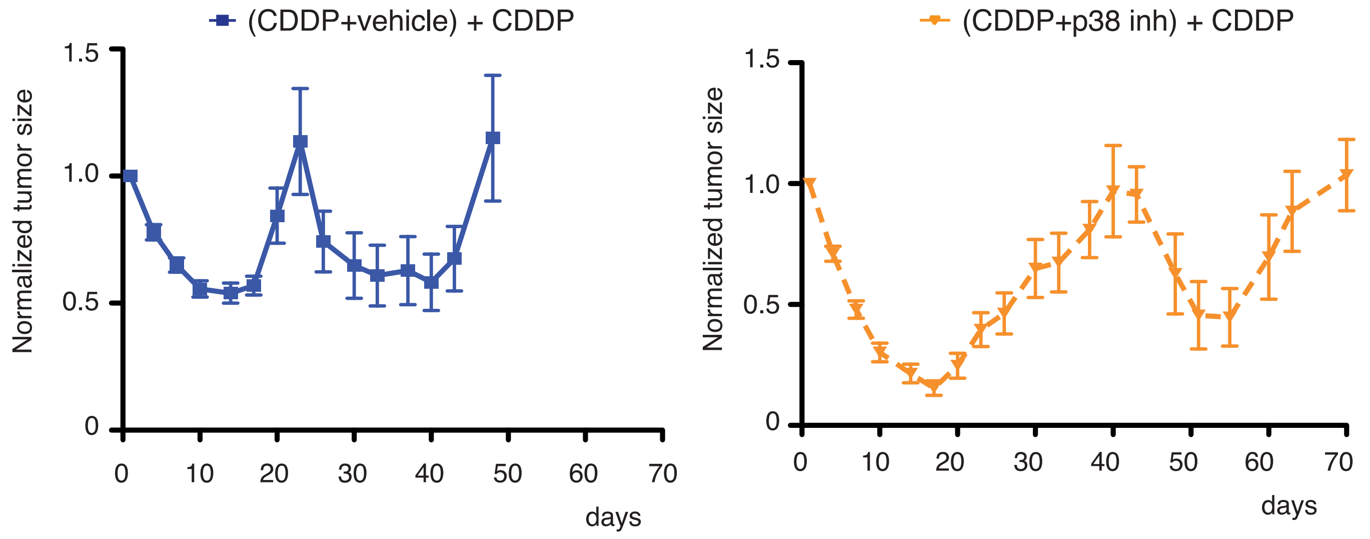

Figure S11.
MMTV-PyMT female mice with breast tumors of about 200 mm^3 in volume were treated with a single-dose of cisplatin (CDDP) followed by daily administration of PH-797804 (PH, 10 mg/Kg) or vehicle for 15 days and tumor growth was monitored until tumors reached again 200 mm^3. Then, a second injection of CDDP was administered to the mice and tumor growth was monitored for up to 70 days . Tumor size was measured at the indicated times and normalized relative to the original size of each tumor when the treatment began. The graph compiles the results of three independent experiments, in which at least four mice per condition were used.

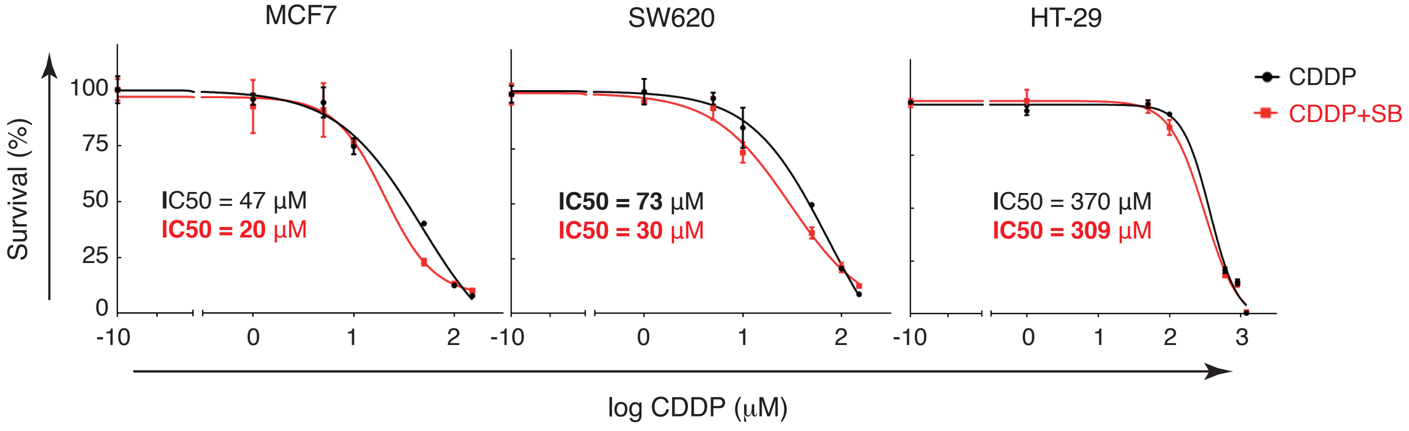

Figure S12.
MCF7, SW620 and HT-29 cells were treated with increasing concentrations of cisplatin (CDDP) with or without SB203580 (SB, 10 µM) for 24 h. Cell viability was measured using the MTT assay. The percentages of viable cells relative to non-treated cells were calculated.
