# Supplementary material for: Inhibition of p38 MAPK sensitizes tumour cells to cisplatin-induced apoptosis mediated by reactive oxygen species and JNK
Source: EMBO Mol Med. 2013 Sep 24;5(11):1759–74. doi: 10.1002/emmm.201302732 (PMC3840490; doi:10.1002/emmm.201302732)
Supplement: Supplementary file 3 [file emmm0005-1759-SD3.pdf]

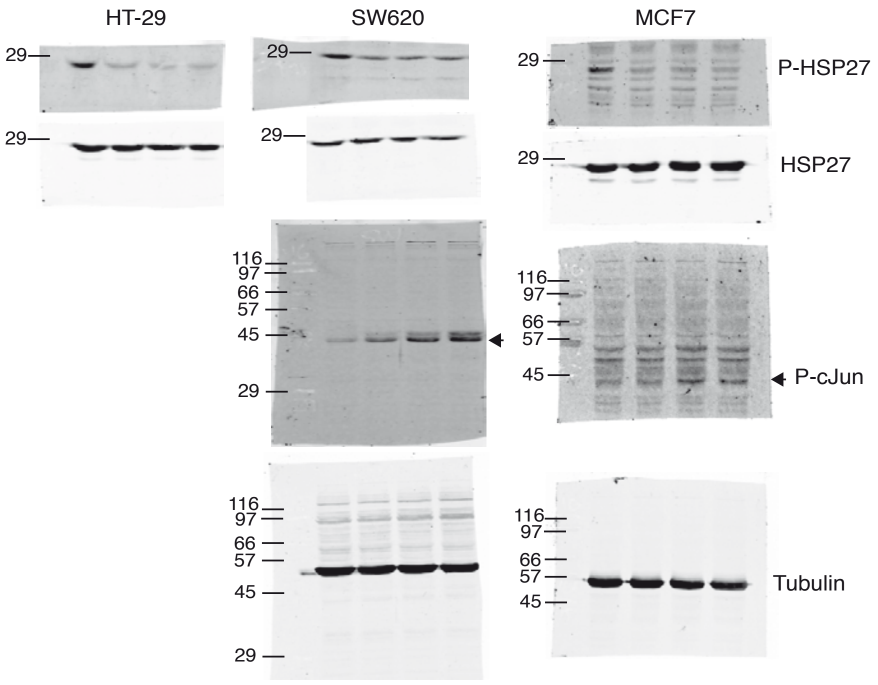

Figure 1D

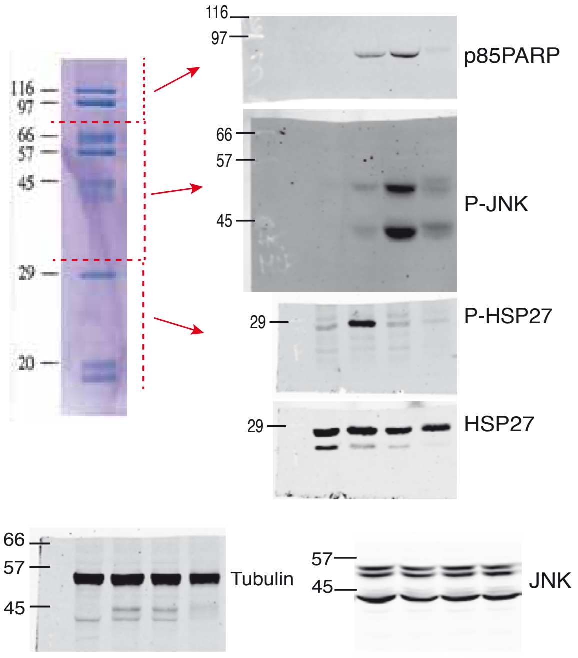

Figure 1E

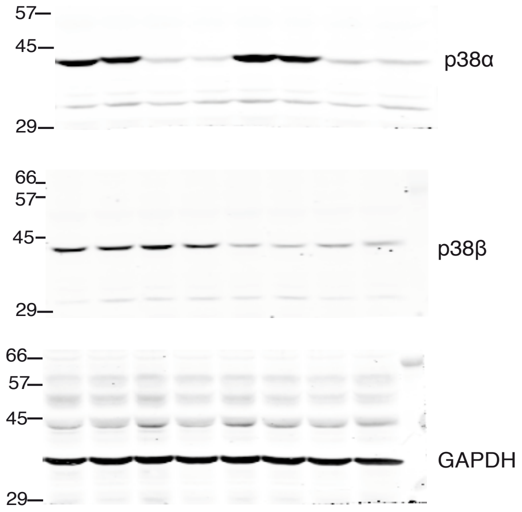

Figure 2C

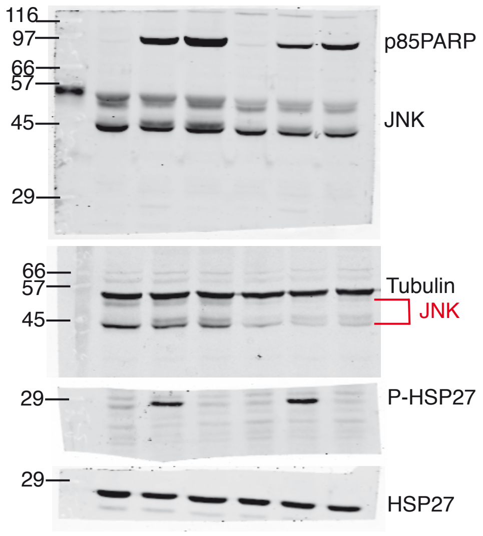

Figure 2E

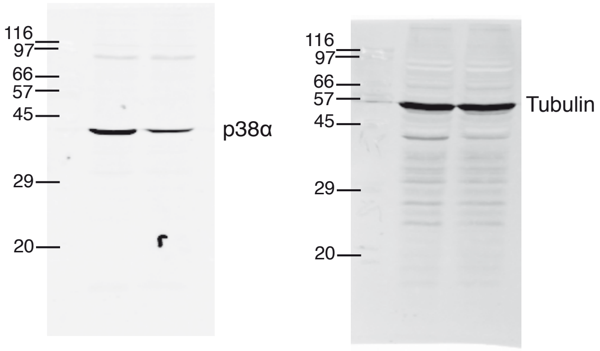

Figure 3B

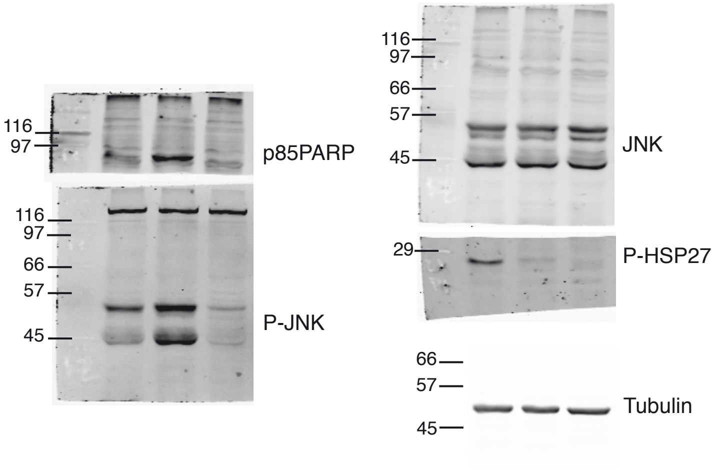

Figure 3C

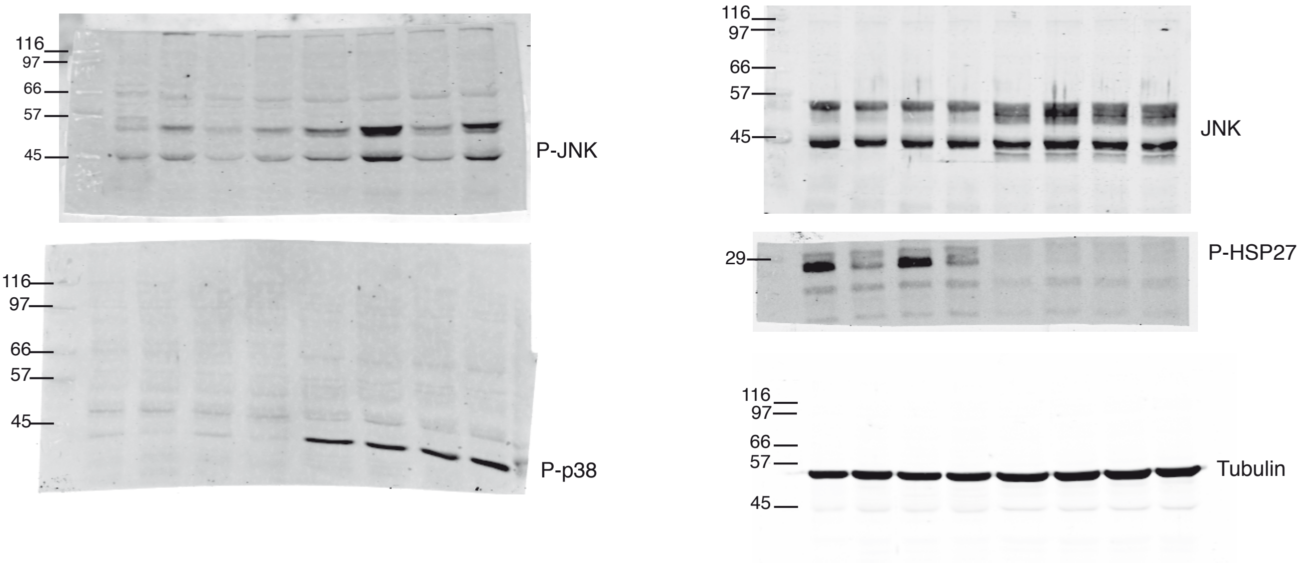

Figure 4B

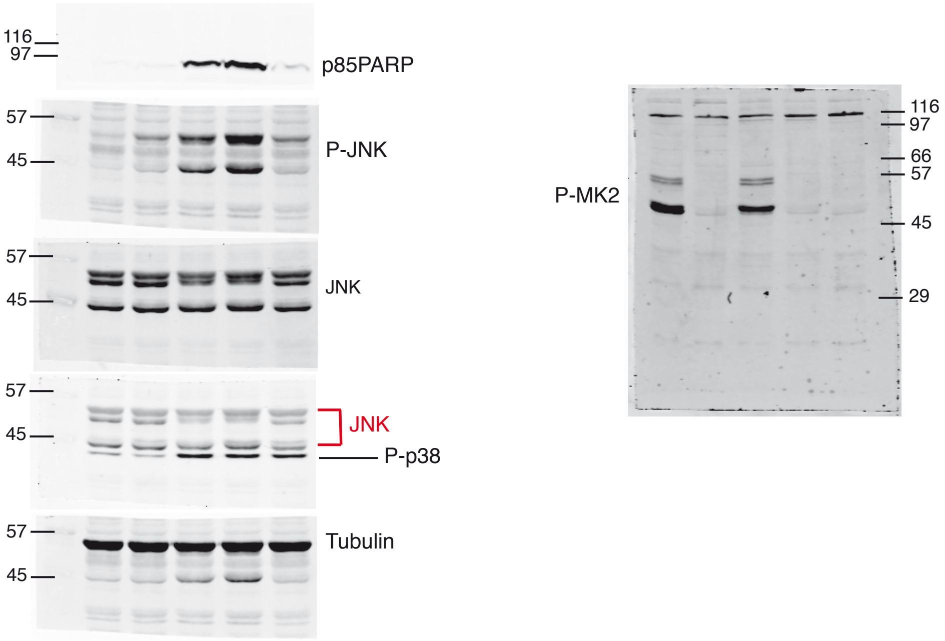

Figure 4C

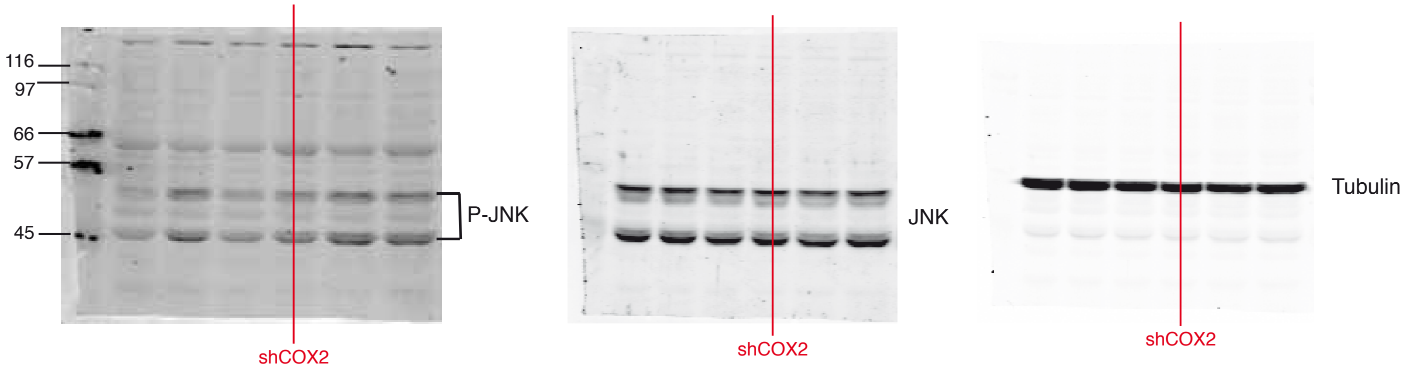

Figure 5B

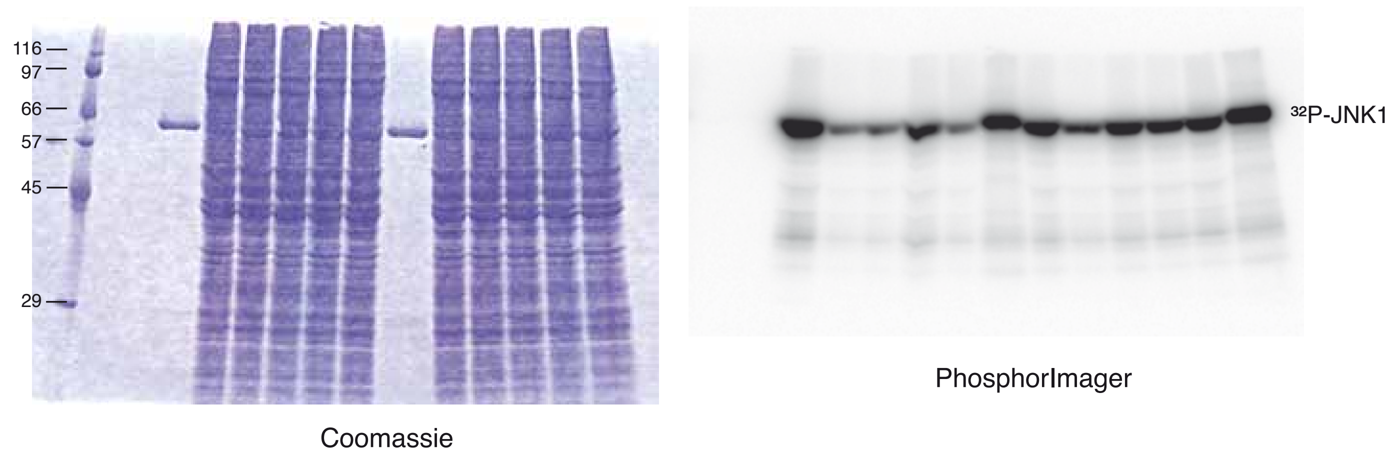

Figure 6A

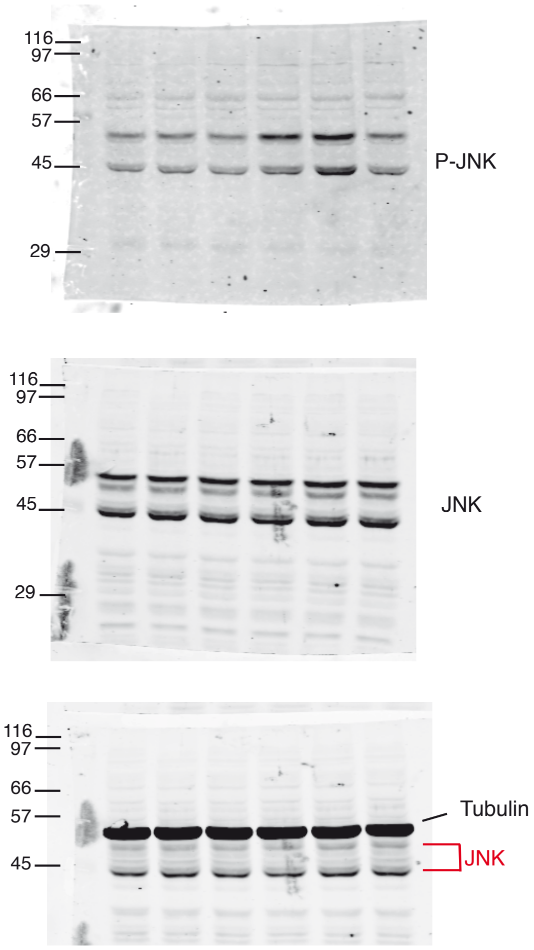

Figure 6C

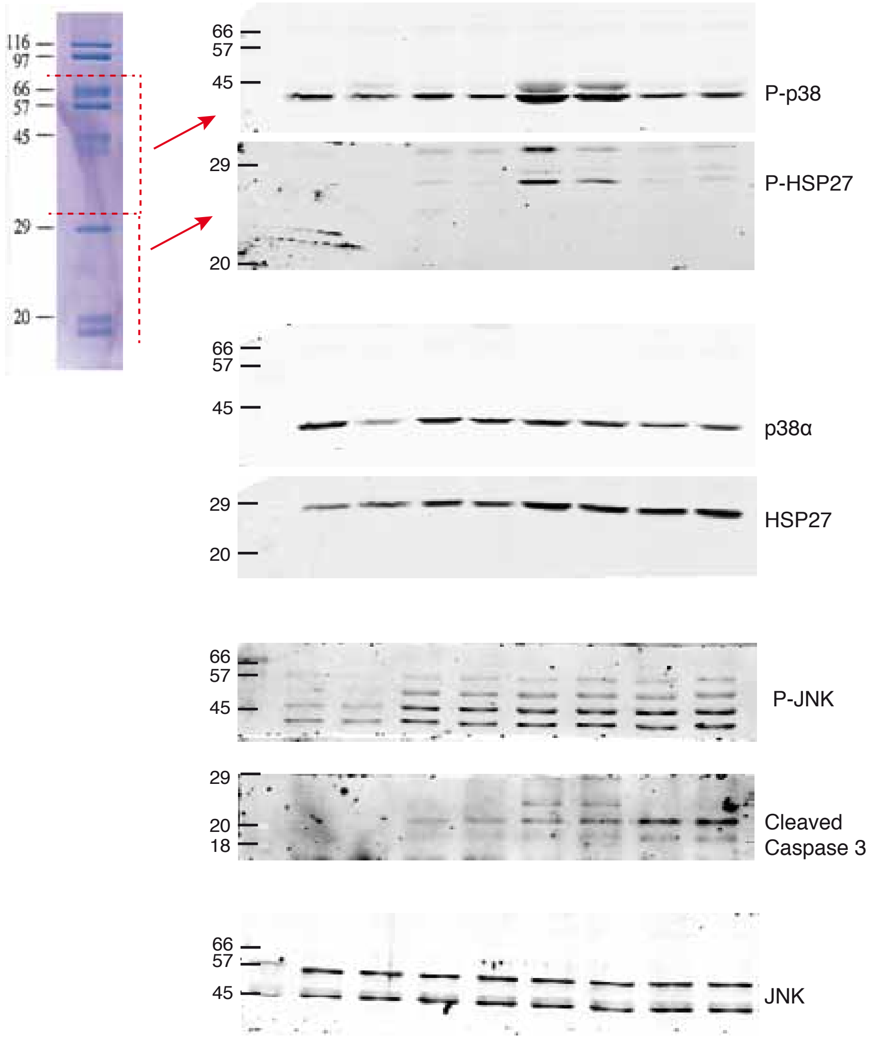

Figure 7C

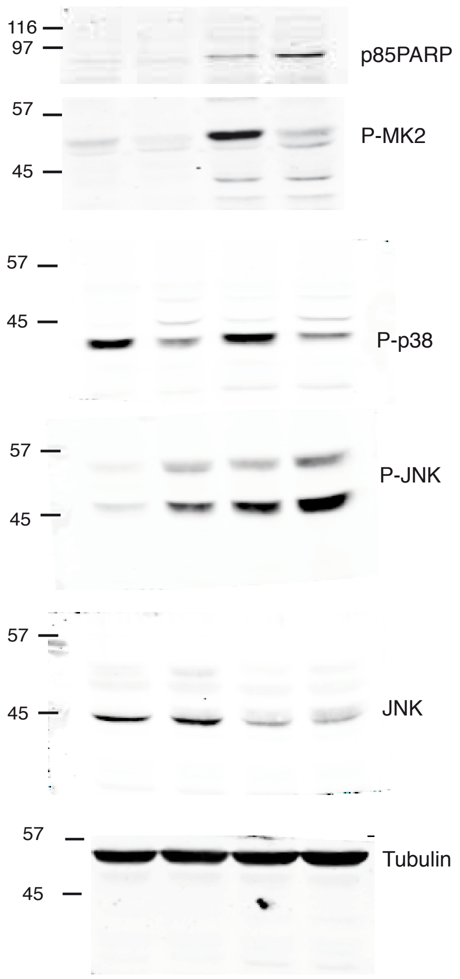

Figure S1C

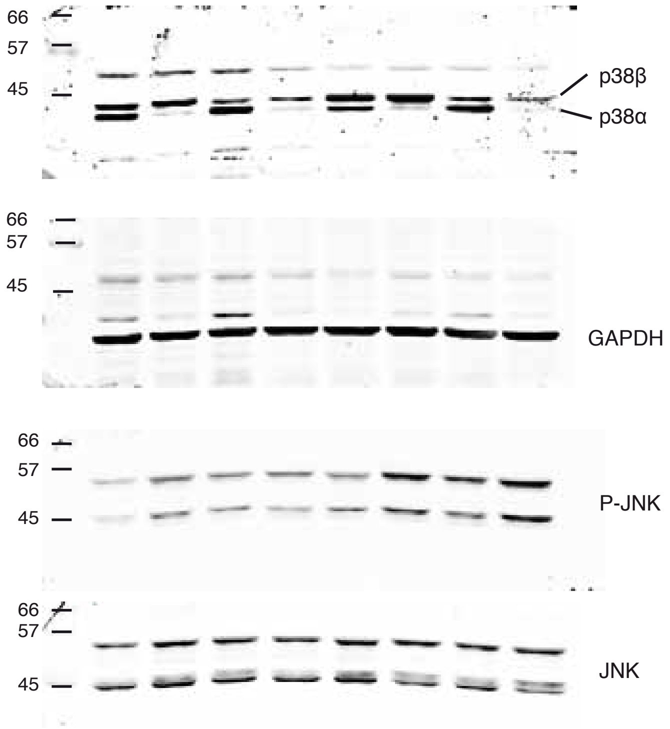

Figure S2A

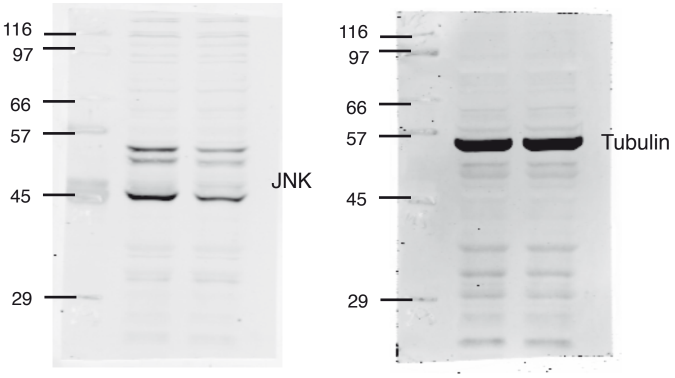

Figure S2B

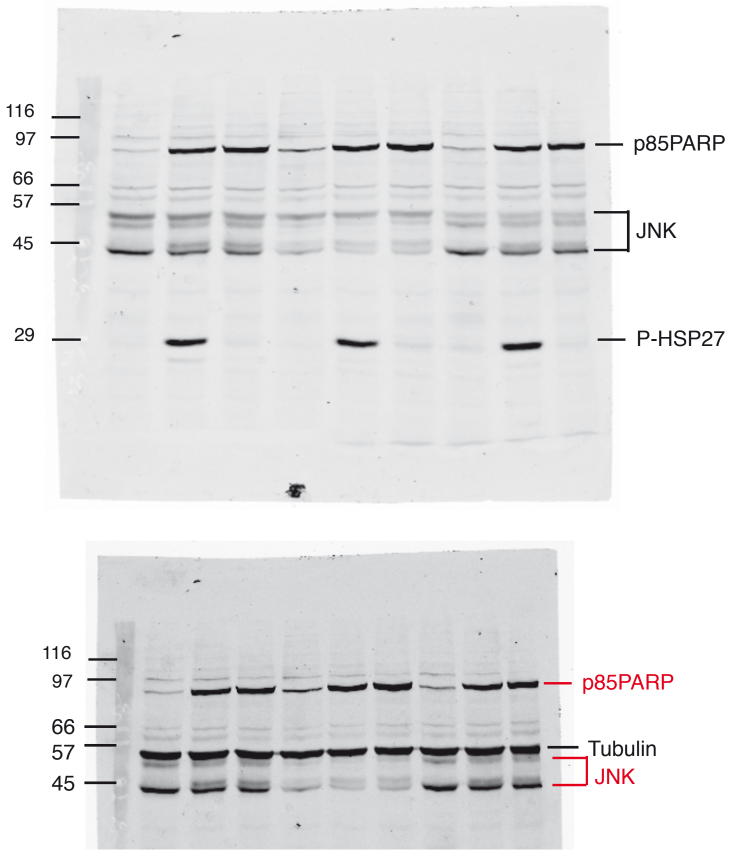

Figure S2C

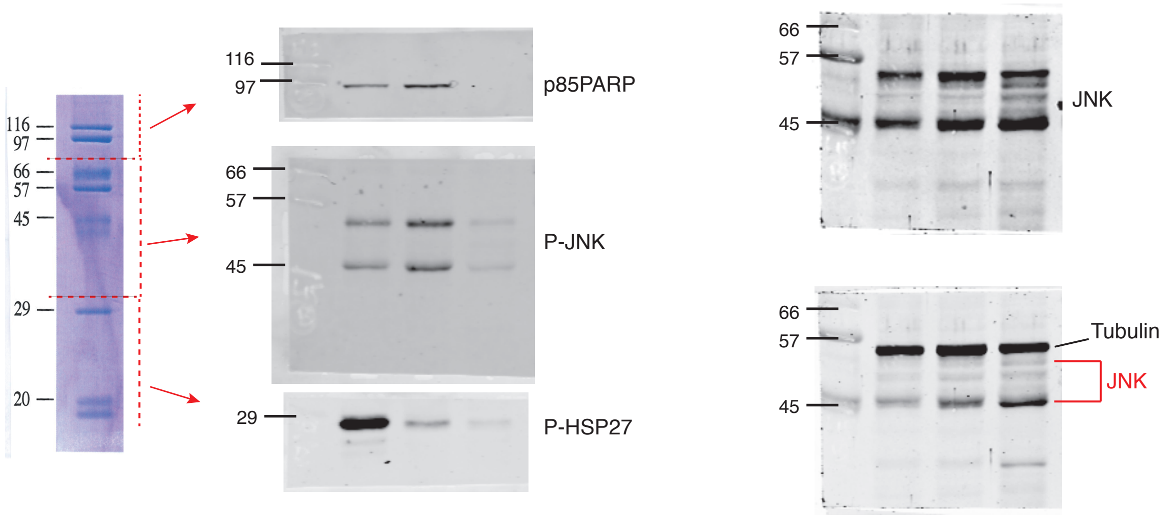

Figure S3C

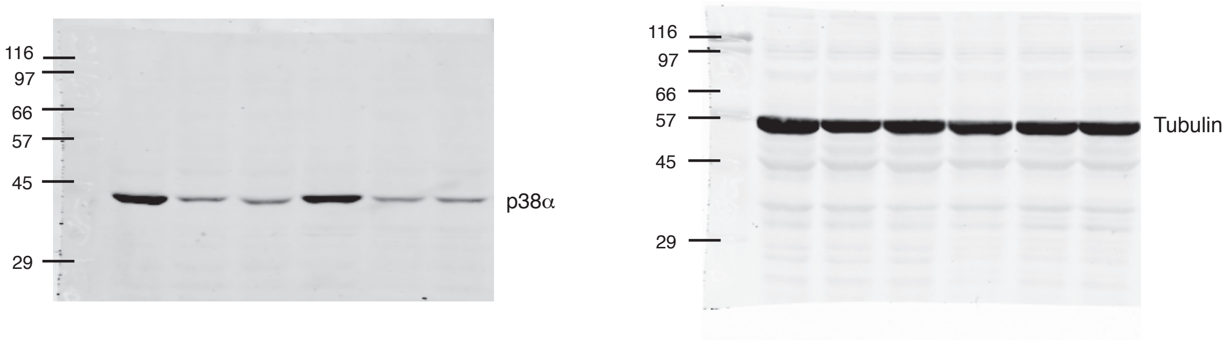

Figure S4B

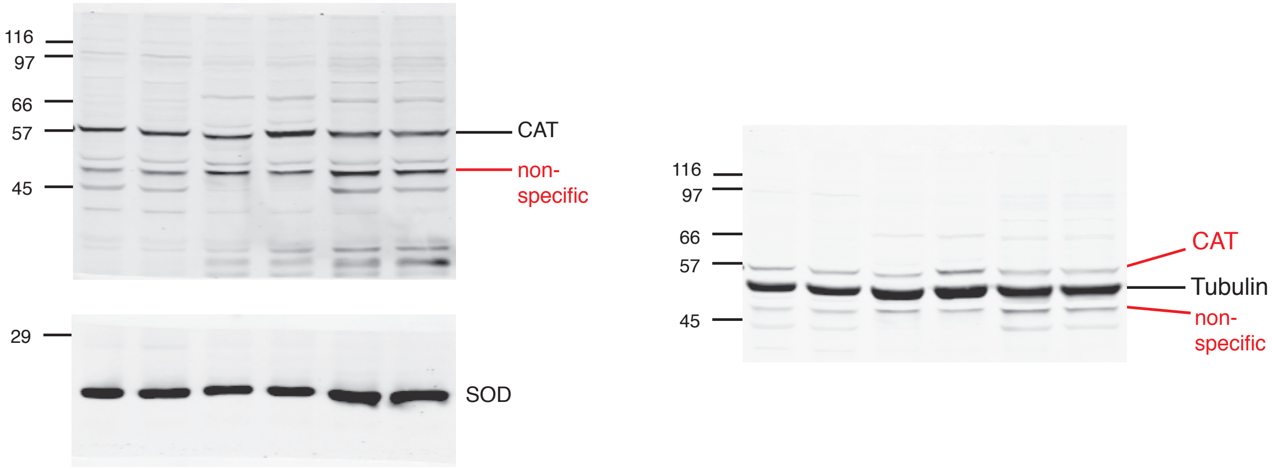

Figure S6

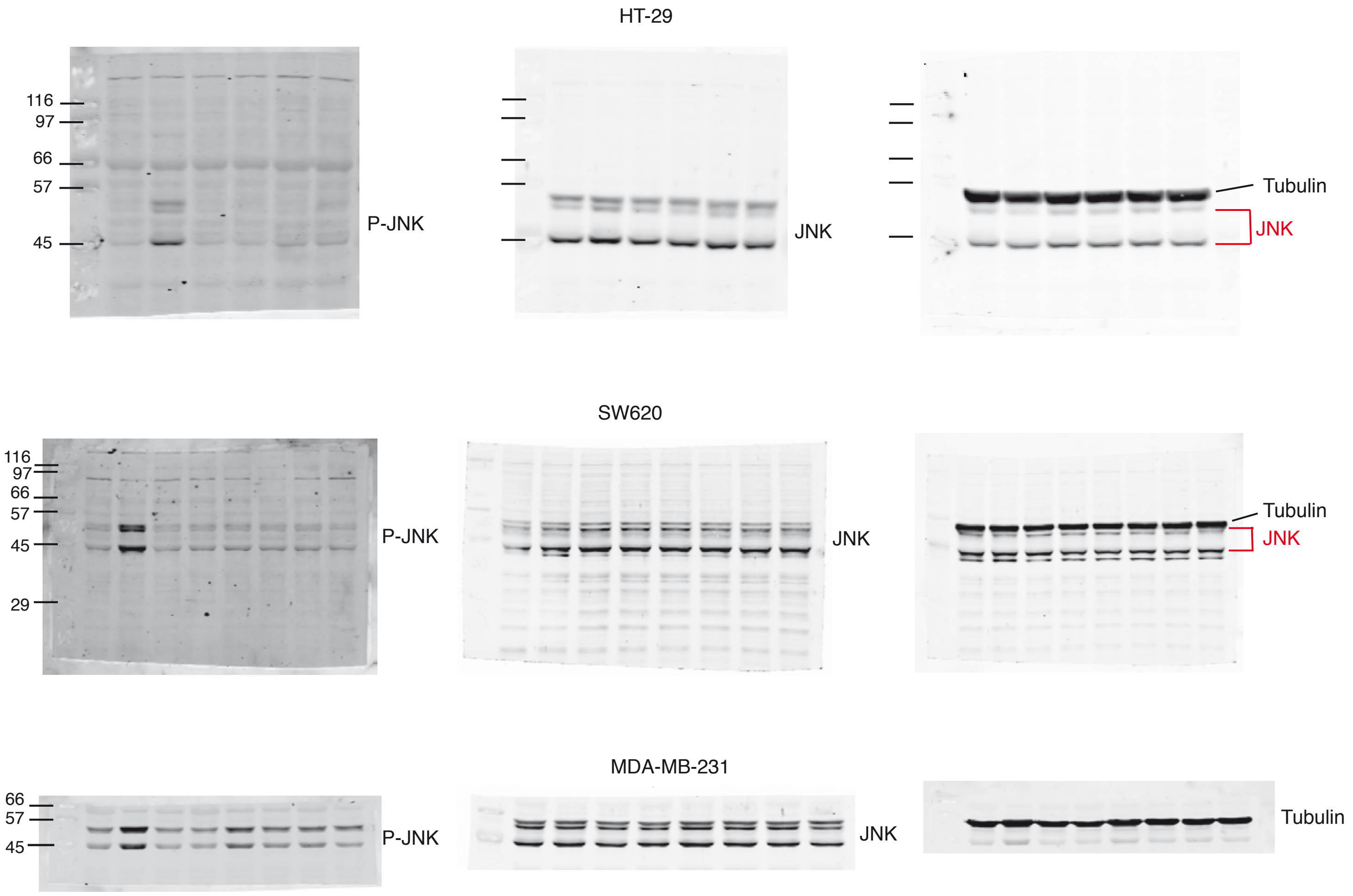

Figure S7

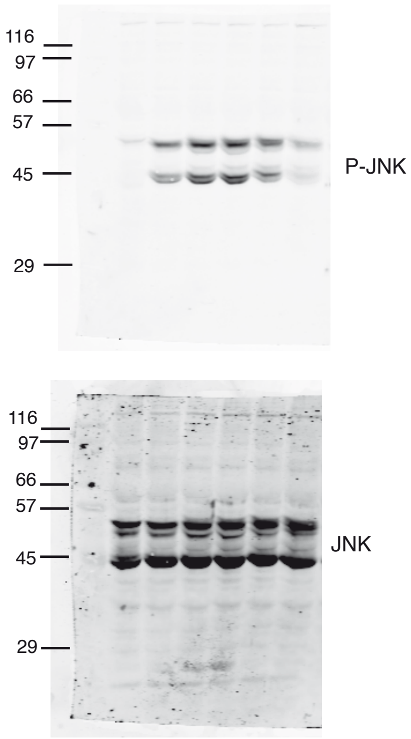

Figure S8A

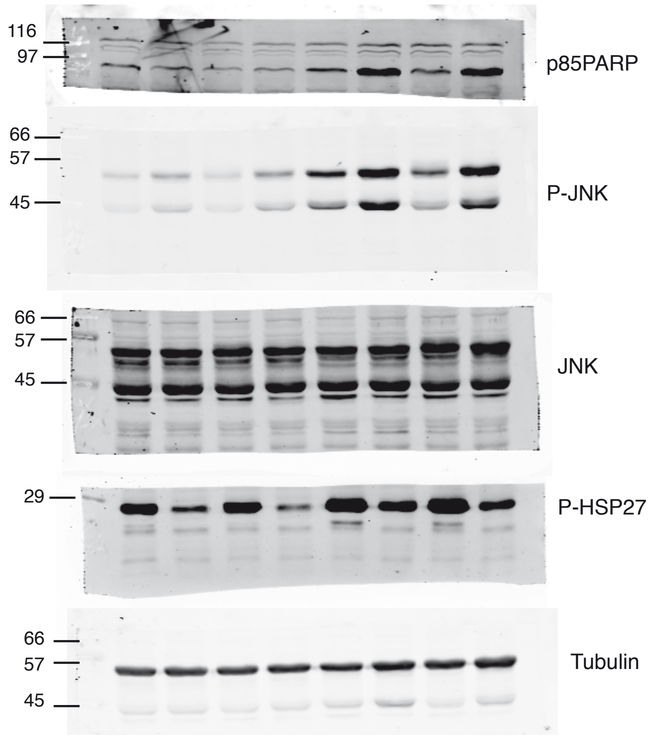

Figure S8B
